# PCL/Gelatin/Graphene Oxide Electrospun Nanofibers: Effect of Surface Functionalization on In Vitro and Antibacterial Response

**DOI:** 10.3390/nano13030488

**Published:** 2023-01-25

**Authors:** Nazirah Hamdan, Wan Khartini Wan Abdul Khodir, Shafida Abd Hamid, Mohd Hamzah Mohd Nasir, Ahmad Sazali Hamzah, Iriczalli Cruz-Maya, Vincenzo Guarino

**Affiliations:** 1 Department of Chemistry, Kulliyyah of Science, International Islamic University Malaysia Kuantan Campus, Bandar Indera Mahkota, Kuantan 25200, Pahang, Malaysia; 2 SYNTOF, Kulliyyah of Science, International Islamic University Malaysia Kuantan Campus, Bandar Indera Mahkota, Kuantan 25200, Pahang, Malaysia; 3 Department of Biotechnology, Kulliyyah of Science, International Islamic University Malaysia Kuantan Campus, Bandar Indera Mahkota, Kuantan 25200, Pahang, Malaysia; 4 Institute of Science, Universiti Teknologi MARA, Shah Alam 40450, Selangor, Malaysia; 5 Institute of Polymers, Composites and Biomaterials, National Research Council of Italy, Mostra d’Oltremare Pad.20, V.le J.F.Kennedy 54, 80125 Naples, Italy

**Keywords:** polycaprolactone, nanofibers, electrospinning, graphene oxide, plasma treatment, antibacterial

## Abstract

The emergence of resistance to pathogenic bacteria has resulted from the misuse of antibiotics used in wound treatment. Therefore, nanomaterial-based agents can be used to overcome these limitations. In this study, polycaprolactone (PCL)/gelatin/graphene oxide electrospun nanofibers (PGO) are functionalized via plasma treatment with the monomeric groups diallylamine (PGO-M1), acrylic acid (PGO-M2), and *tert*-butyl acrylate (PGO-M3) to enhance the action against bacteria cells. The surface functionalization influences the morphology, surface wettability, mechanical properties, and thermal stability of PGO nanofibers. PGO-M1 and PGO-M2 exhibit good antibacterial activity against *Staphylococcus aureus* and *Escherichia coli*, whereas PGO-M3 tends to reduce their antibacterial properties compared to PGO nanofibers. The highest proportion of dead bacteria cells is found on the surface of hydrophilic PGO-M1, whereas live cells are colonized on the surface of hydrophobic PGO-M3. Likewise, PGO-M1 shows a good interaction with L929, which is confirmed by the high levels of adhesion and proliferation with respect to the control. All the results confirm that surface functionalization can be strategically used as a tool to engineer PGO nanofibers with controlled antibacterial properties for the fabrication of highly versatile devices suitable for different applications (e.g., health, environmental pollution).

## 1. Introduction

The rise of antibiotic-resistant bacteria has become a major concern in medical and public health, particularly in controlling the spread of infectious diseases. According to the Centre for Disease Control, CDC (2019), antibiotic-resistant bacteria have caused at least 2,868,700 infections with 35,900 reported deaths [1]. The overuse and misuse of antibiotics contribute to the development of these superbugs [2,3,4,5]. Thus, infectious diseases are harder to treat as antibiotics become less effective, causing damage to the immune system and leading to treatment failure [6,7]. This has led to the development of other antimicrobial agents, such as carbon nanomaterials and anti-biofilm gel, as alternatives to antibiotics to prevent bacteria resistance [8,9,10]. Carbon nanomaterials such as graphene oxide (GO) have been widely used in biomedical applications due to their biocompatibility and stability [11,12,13]. Graphene oxide is composed of linked sp^2^-hybridized carbon atoms, with several oxygen functional groups, hydroxyl, carbonyl, and epoxy, attached to its basal plane, which increases its reactivity [14,15,16]. Previous studies have demonstrated a strong antibacterial activity of chemically modified GO against Gram-positive and Gram-negative bacteria and their capacity to cover bacterial cells and thus alter cell morphology [17,18,19]. Despite its remarkable antimicrobial activity, there are increasing concerns regarding GO’s toxicity to human cells. Cytotoxicity studies found that GO increases cell oxidative stress, leading to DNA damage, even at low concentrations (5 and 10 wt%), and further reduces cell viability and proliferation [20,21]. In comparison, GO-loaded polymer nanofibers showed no cytotoxicity effects on neural and keratinocyte cells at concentrations of up to 1.5 wt% [22]. These findings indicate that the toxicity of GO is dose dependent. Therefore, it is vital to control its concentrations within a therapeutic range for human use. 

In recent years, great efforts have been made in the fabrication of nanostructured scaffold materials with antimicrobial properties [23]. In particular, the electrospun nanofibers’ properties, such as the fiber diameter, alignment, high surface area to volume, high porosity, and interconnected pores, can be tailored to not only deliver antimicrobial agents locally to an infected area but also kill bacteria [24,25,26,27,28,29,30]. In some cases, natural and synthetic polymer scaffolds are combined in a design to further improve the biocompatibility, mechanical strength, wettability, and degradation rates [31,32,33]. Polycaprolactone (PCL), a synthetic polymer approved by the Food and Drug Administration (FDA), is frequently used in biomedical applications due to its low-cost materials with high spinnability, good mechanical properties, and excellent biodegradability compared to other polymers [34,35,36]. However, the slow degradation rate and hydrophobic nature of PCL limit its ability to mobilize and release therapeutic agents. Another material, gelatin, a natural hydrophilic polymer obtained through the denaturation of collagen, is biocompatible, biodegradable, non-toxic, and non-antigenic [37,38,39,40,41], but it has insufficient mechanical strength and is difficult to electrospin. Hence, to overcome these issues, the two materials can be blended to obtain nanofibers with better physicochemical properties [42,43,44]. PCL/gelatin blends have been shown to improve the mechanical properties, hydrophobicity, and enhanced thermal stability of nanofibers compared to pure PCL and gelatin [45,46,47]. 

Over the years, the electrospinning technique has been used to fabricate polymeric nanofibers for various applications. The unique characteristics of nanofibers, such as their high surface area, high porosity, and small pore size, make them suitable for biomedical applications [48,49,50,51]. Nanofibers can also be attached to specific functional groups, such as carboxylic acid, amines, and aldehydes, via plasma surface treatment. The different structural and chemical properties possessed by the functional groups, such as polarity, acidity, and charges, can highly influence the surface interaction between nanofibers and bacteria cells [52,53,54,55,56]. For instance, the surface wettability of nanofibers can influence their hydrophobicity [57], potentially leading to the strong adhesion of hydrophobic bacteria such as *S. aureus* to their surfaces [58,59]. In contrast, a hydrophilic surface can effectively inhibit the adhesion of the bacteria, as the surface bonding between the bacteria and nanofibers is weak [60]. Positively charged cationic nanofibers have been shown to increase antimicrobial potency, as they can attract negatively charged bacteria cells [61,62,63]. Post-electrospinning surface treatments can be optimized to introduce suitable moieties on the surface of nanofibers using several approaches, including wet chemical functionalization, covalent grafting, physical adsorption, and plasma treatment [64,65,66,67,68]. Among these methods, plasma treatment is favorable, as it is the fastest method for surface functionalization with a high stability effect [69,70]. 

Herein, GO is synthesized and blended with PCL/gelatin nanofibers, functionalized by monomer moieties (i.e., diallylamine, acrylic acid, and *tert*-butyl acrylate), and optimized to validate its antibacterial activity against *S. aureus* and *E. coli.* The surface functionalization is realized with the support of plasma treatment. The functionalized nanofibers are characterized by scanning electron microscopy (SEM), Fourier transform infrared spectroscopy (FTIR), X-ray photoelectron spectroscopy (XPS), and thermogravimetric analysis (TGA). The contact angle is measured via the drop method to determine the wettability of the nanofibers. In addition, the antibacterial activity and cell viability of the functionalized nanofibers are evaluated.

## 2. Materials and Methods

### 2.1. Materials 

Polycaprolactone, PCL (MW = 80 kDa), gelatin from bovine skin (CAS 9000-70-8), and tert-butyl acrylate were purchased from Sigma Aldrich, Milan, Italy. Graphite, sulfuric acid, phosphoric acid, hydrogen peroxide, hydrochloric acid, chloroform, methanol, 1,1,1,3,3,3-hexafluoro-2-propanol (HFIP), acrylic acid, diallylamine, nutrient broth, nutrient agar, and sodium chloride (NaCl) were purchased from Merck, Darmstadt, Germany. Potassium permanganate was obtained from Bendosen Laboratory Chemicals, Johor Malaysia. The Live/Dead Baclight Bacterial Viability Kit (L7007) was purchased from Thermo Fisher Scientific, Bleiswijk, The Netherlands. The bacterial strains of *S.aureus* (ATCC 25293) and *E.coli* (ATCC25922) were received from the Department of Biotechnology, Kulliyah of Science, International Islamic University, Pahang, Malaysia. 

### 2.2. Synthesis and Characterization of Graphene Oxide (GO)

#### 2.2.1. Synthesis of GO

Graphene oxide (GO) was synthesized using the modified Hummers method with slight modifications [71,72]. Briefly, 3 g of graphite powder was added to a mixture of sulfuric acid (H_2_SO_4_) and *ortho*-phosphoric acid (H_3_PO_4_) at a 9:1 (*v/v*) ratio and stirred for 2 h until homogeneous in an ice bath. Then, 6 g potassium permanganate (KMnO_4_) was added slowly with continuous stirring for 1 h. The solution was sonicated for 15 min until homogeneous. Next, 80 mL of deionized water was added and the solution was stirred at 40 °C for 30 min. Then, the solution was stirred for another 40 min at 80 °C to speed up the rate of reaction. The solution was left to cool to room temperature before 100 mL of hydrogen peroxide (H_2_O_2_) was added to the solution to stop the reaction and the solution was stirred vigorously. The obtained solution was separated by decantation, followed by several steps of centrifugation for 7 min at 5000 rpm. The solid residue was re-suspended three times in 40 mL of 20 % (*v/v*) hydrochloric acid (HCl) and another three times in distilled water and further centrifuged for purification. The obtained graphene oxide was kept at –80 °C and then freeze-dried for 24 h and stored in a desiccator for further analysis.

#### 2.2.2. Characterization of GO

The morphology of the GO sheets was examined using transmission electron microscopy, TEM (Carl Zeiss Libra 120, Oberkochen, Germany). A small amount of GO was dispersed in distilled water and dropped on the grid. The sample was viewed using TEM at 10,000× magnification. The morphological surface features and roughness of the graphene oxide were studied using atomic force microscopy (NX-10, Park Systems, Suwon, Korea) at 5 and 10 µm dimensions. The images obtained were analyzed using XEI software (Version 4.3.1., Park Systems, Suwon, Korea). The possible chemical interactions of the GO samples were analyzed using attenuated total reflection Fourier transform infrared (ATR-FTIR, Perkin Elmer, Waltham, MA, USA), spectroscopy at a spectral range of 600–4000 cm^–1^. The Raman spectra of the GO were measured at room temperature with a DXR Raman microscope at an excitation wavelength of 532 nm and a Raman shift range of 1000–30000 cm^–1^ to study the structural properties of the GO sheets including the layer, disorder, and defect level.

### 2.3. Fabrication of Blended PCL/Gelatin/GO Nanofibers

Briefly, 10% *w/v* PCL was dissolved in chloroform: methanol (3:1 *v/v*) solution and stirred for 24 h at room temperature [73,74]. Meanwhile, 10% *w/w* gelatin was dissolved in HFIP and stirred for 24 h. The PCL/gelatin (PG) solution was mixed at a 1:1 (*v/v*) solvent ratio and stirred for 24 h. Next, 1.0% (*w/v*) of GO, was added to the solution and stirred for another 24 h before electrospinning. The electrospinning of the polymer solution was performed using a custom-built electrospinning setup consisting of KD Scientific Flow Rate apparatus and ES 50P-10W Gamma High Voltage (ES 50P-10W) with pre-optimized parameters at a 0.8 mL/h flow rate, 15 kV voltage, 12 cm tip-to-collector distance with a 20 G (0.91 mm) needle.

### 2.4. Surface Functionalization of Nanofibers

The surface functionalization of the nanofibers was performed by direct current plasma treatment method or glow discharge plasma. This modified the fibers’ surfaces for grafting three different kinds of monomers—diallylamine (M1), acrylic acid (M2), and *tert*-butyl acrylate (M3)—with a recognized ability to influence bacteria attachment to polymer surfaces [75,76]. Three different monomers were introduced to investigate the effects of different functional groups on the wettability of the nanofibers, as well as the bacterial attachment ability of each functional group. A custom-built plasma system was set up consisting of a two-electrode cathode (voltage) and an anode (ground collector), which operated with atmospheric-pressure air. Nitrogen gas was supplied through a glass tube to create a plasma discharge. The nanofiber samples were placed on the anode near the plasma discharge. The monomers’ vapors were sprayed using an automatic mist sprayer in between the cathode and anode at a 10 mm distance, 10 L/min flow rate, and 15 kV voltage for 30 s. The plasma-treated samples were labeled PGO-M1, PGO-M2, and PGO-M3 respectively.

### 2.5. Characterization of Nanofibers

The surface morphologies of the nanofibers before and after treatment were examined using scanning electron microscopy, SEM (Carl Zeiss S.E Asia/ZEISS Evo 50, Oberkochen, Germany). Briefly, a small section of a nanofiber sample was mounted on a brass stub using double adhesive tape and sputter-coated with a gold–palladium mixture under vacuum using a Sputter Coater (Leica EM SCD005, Wetzlar, Germany). The samples were analyzed at 2000× magnification. The diameters were measured from 100 nanofibers randomly using ImageJ Software (Version 1.52a, National Institute of Mental Health, Bethesda, MD, USA). The functional groups of the nanofiber samples were identified using ATR-FTIR (Perkin Elmer, Waltham, MA, USA) at a spectral range of 600–4000 cm^–1^. The wettability of the nanofibers was determined by water contact angle measurements using the drop method, where deionized water was dropped onto the nanofibers’ surfaces. The measurements were performed in triplicate and the average contact angle was determined. The elemental scan of XPS was performed to study the surface chemistry of the nanofibers using a Kratos Axis Ultra DLD. The spectra were scanned at the range of 0–1200 eV and survey scans of C1s, O1s, and N1s were obtained. The atomic ratios were analyzed and calculated using Casa XPS Software (Version. 2.3.25). The thermal stability of the nanofibers was examined by thermogravimetric analysis using 2–5 mg nanofibers at a temperature range of 35–500 °C and a heating rate of 10 °C/min under a nitrogen atmosphere. The mechanical properties of the functionalized nanofibers were measured by an axial tensile testing machine (Omron ZR-RX25 Ver1.06). The sample was cut into 20 × 20 mm and mounted between two clamps. The gauge length was measured over time until the total failure of the sample at room temperature. The stress–strain curve of the sample was obtained using Sigmaplot software (ver.14.5).

### 2.6. Antibacterial Assay

#### 2.6.1. Plate-Counting Assay

The E. coli and S. aureus bacteria were cultured overnight for five consecutive days to obtain a single colony of bacteria. The isolated colony was then transferred into a 10 mL nutrient broth of a 0.5 McFarland standard. The optical density of OD 600 nm was obtained at 0.0870. Next, 1000 µL of the bacteria suspension was transferred into 9 mL fresh broth to make up a total of 10 mL of bacteria suspension. The nanofiber sample was sterilized under a UV light for 30 min before being added to the bacterial suspension and incubated at 37 °C for 24 h.

After 24 h, the sample was taken out and rinsed three times with sterile phosphate buffer saline (PBS, pH 6.8) to remove the excess suspension and unattached bacteria. The sample was immersed in a 10 mL sterile PBS solution. The suspension was further diluted by transferring 100 µL of the cultured PBS solution to a 9.9 mL sterile PBS solution to make 10^–2^, 10^–4^, and 10^–6^ serial dilutions. Each tube was vortexed for 1 min and incubated at 37 °C for 15 min before being diluted. Afterward, 100 µL of the 10^–6^ solution was transferred to the nutrient agar plate and spread using a sterile cotton swab. The agar was incubated at 37 °C for 24 h and the colonies formed on the plate were counted using the Formula (1),

(1) $$Colony\;counts\;\left(\frac{CFU}{mL}\right)=\frac{Total\;number\;of\;colonies\;\times \;dilution\;factor}{Volume\;of\;culture\;plate\;\left(mL\right)}$$

where the dilution factor is 10^–6^ and the volume of the culture plate is 2 mL [77].

#### 2.6.2. Bacterial Attachment Study

The live/dead bacteria cell assay was performed to study the bacterial attachment on the different surface chemistries of the plasma-treated nanofibers. Briefly, the bacteria were sub-cultured for five consecutive days to obtain a single colony. The single colony was transferred from the agar plate to a 5 mL sterile nutrient broth and incubated at 37 °C for 24 h. The OD_600nm_ of the bacterial suspension was taken using a UV-Vis spectrometer at 600 nm (OD_600nm_ = 0.3). Then, 100 µL of the broth culture was spread on the agar plate using a sterile cotton swab. The plate was incubated at 37 °C for 18 h until the entire plate was covered with bacteria biofilm. After 18 h, the sterile nanofiber sample was incubated on the plate facing down for another 1 h at 37 °C. After the incubation, the sample was rinsed with a sterile 0.85% NaCl solution to remove unattached bacteria for further staining and characterization [78]. 

The staining solutions were prepared according to the manufacturer’s instructions. Briefly, two dyes, SYTO 9 dye with 1.67 mM/Propidium iodide and 1.67 mM (component A) and SYTO 9 dye with 1.67 mM/Propidium iodide and 18.3 mM (component B), were used for staining. The two components (A and B) were mixed thoroughly at a 1:1 ratio in a microfuge tube. Then, 6 µL of the pre-mixed staining solution was added into 2 mL of sterile 0.85% NaCl solution. Afterward, the nanofiber sample was immersed in the staining solution for 30 min at 37 °C in a dark room. The staining was removed and the sample was rinsed once with a 0.85% NaCl solution and viewed under a fluorescence microscope (Olympus BX53) at 40× magnification. Selected filters were used: UMWB2 (excitation at 460–490 nm) and UMWG2 (excitation at 510–550 nm).

The morphological characteristics of the attached bacteria were studied using SEM analysis. Briefly, the samples were incubated with bacteria using the same method described in Section 2.6.1. After incubation, the samples were washed thrice with a sterile 0.85% NaCl solution to remove any unattached bacteria. Then, the samples were immersed in a 2 mL formaldehyde solution in a PBS solution (3.4% *v/v*) for 30 min for bacterial fixation [78]. Afterward, the samples were rinsed once with 0.85% NaCl and immersed in three subsequent ethanol aqueous solutions at increasing concentrations (50, 70, 90%) for dehydration. Then, the samples were mounted on the stub and further coated for viewing.

### 2.7. In Vitro Tests

To evaluate the biological response of the nanofibers, L929 cells derived from mice (Sigma Aldrich, St. Louis, MO, USA) were used. The L929 cells were cultured in 75 cm^2^ cell culture flasks containing Dulbecco’s Modified Eagle Medium (DMEM, Sigma-Aldrich, St. Louis, MO, USA) supplemented with 10% fetal bovine serum (FBS, Sigma-Aldrich, St. Louis, MO, USA), 2 mM L-glutamine, and antibiotic solution (streptomycin 100 μg/mL and penicillin 100 U/mL, Sigma-Aldrich, Milan, Italy). The cell cultures were incubated in a 100% humidified environment at 37 °C in 95% air and 5% CO_2_. Before the biological assays, the nanofibers were placed in a 96-well culture plate and sterilized with 70% ethanol solution for 30 min. After sterilization, the nanofibers were rinsed with PBS three times and air-dried.

The cell viability of L929 onto nanofibers was analyzed after 1, 3, 7, and 14 days of culture. First, the viability was verified by the XTT assay (Roche) based on the cleavage of the yellow tetrazolium salt XTT to form an orange formazan dye by metabolically active cells. The concentration of the formazan product is directly proportional to the number of metabolically active cells. The L929 cells were seeded at 5 × 10^3^ cells/well onto the nanofibers. During the experimental time, the culture medium was exchanged every third day. After the prescribed time points, the culture media was changed by 100 μL of fresh medium containing 50 μL of XTT working solution and incubated for 4 h under standard conditions. Then, the supernatant was removed and placed in a microplate reader (ELISA) and the absorbance was quantified by spectrophotometry (Wallac Victor3 1420, PerkinElmer, Boston, MA, USA) at 450 nm. 

### 2.8. Statistical Analysis

All data in this study are expressed as average ± standard deviation. Data analysis was performed using OriginPro Software (Version 8.5.0, Northampton, Massachusetts, USA), and CasaXPS (Version 2.3.25PR1.0). ImageJ Software (Version 1.52a, National Institute of Mental Health, Bethesda, MD, USA) was used to measure the average fiber diameters. For the cell viability tests, the results are presented as the mean ± standard error (n = 3). Analysis of variance (ANOVA) with Tukey’s post hoc test was used to detect the differences between the groups. A value of *p* < 0.05 was considered to determine the statistically significant differences.

## 3. Results and Discussion

### 3.1. Characterization of GO

A major recent study of carbon materials, i.e., nanotubes, nano blades, and nano spikes, concentrated on the development of an antibiotic-independent treatment for antibiotic-resistant bacteria [79,80,81]. The antimicrobial activity of carbon materials such as graphene oxide (GO) is highly dependent on its properties including the sheet size, concentration, number of layers, and density of functional groups. GO with a small sheet size, high density of wrinkles and edges, high thickness and surface roughness, as well as large number of hydrophilic oxygen-containing groups (i.e., epoxy, carboxyl, and hydroxyl), has proven to be effective in killing bacterial strains [17,19,82]. The specific requirements of GO properties can be controlled during its synthesis [83]. GO can be synthesized using various established methods such as the Brodie, Staudenmaier, or Hummers methods [84,85]. These methods involve the oxidation of graphite by strong acids and oxidants such as phosphoric acid, sulfuric acid, nitric acid, potassium permanganate, and potassium chlorate [86,87]. The Hummers method is commonly used as it is fast, easy, and can produce GO with higher oxidation and hydrophilic properties [88,89]. 

**Figure 1:**
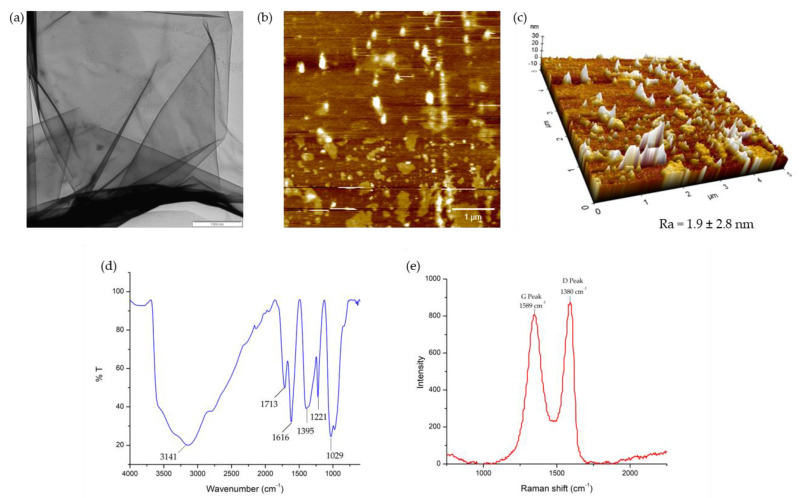
(**a**) TEM image of GO nanosheets at 10,000 magnification (scale bar: 1000 nm); (**b**) 2D AFM lateral images of GO at 5 µm (scale bar: 1 µm); (**c**) 3D AFM lateral images of GO at 5 µm; (**d**) ATR-FTIR spectrum of GO nanosheets; (**e**) Raman intensity of GO nanosheets.

In this study, the GO was synthesized from pure graphite using a modified Hummers method as a green approach to prevent the release of toxic gases [90,91,92]. The surface morphology and properties of GO were studied using TEM, AFM, FTIR, and Raman spectroscopy. The TEM (Figure 1a) revealed thin, wrinkled, highly transparent, and multi-layer sheets of GO [93,94,95]. The average size of the GO sheets obtained was 133 ± 82 nm. The AFM topographic images (Figure 1b,c) show the GO sheets with sharp surfaces with several spikes and roughness (Ra) of 1.9 ± 2.8 nm. The average thickness of the GO sheets of around 3.7 ± 2.2 nm suggests the presence of multi-layer sheets. The morphological results indicate the obtained multi-layer sheets of GO formed were small, with high surface roughness and spike intensity, which may contribute to effective antimicrobial activity [96,97]. 

The FTIR spectra of the GO sheets (Figure 1d) show a band at 3141 cm^–1^, which corresponds to the OH stretching vibration, whereas the band at 1713 cm^–1^ indicates the C=O vibration of the carbonyl group. The absorption band at 1616 cm^−1^ represents the C-C graphitic backbones of GO, whereas the peak at 1395 cm^−1^ represents the C-OH of carboxylic groups. The appearance of the C-O-C epoxy groups is shown at 1221 cm^−1^, and the band at 1029 cm^−1^ represents the C-O vibrations [98,99]. The results confirmed the presence of chemical groups in the GO sheets such as hydroxyl (OH), carbonyl (C=O), carboxyl (COOH), and epoxide (COC) moieties. The Raman spectrum of the GO shows the presence of a characteristic D band at 1380 cm^−1^ due to the disorder of the carbon lattice and a graphitic G band at 1589 cm^−1^ due to the stretching sp^2^ carbon atoms (Figure 1e) [100,101]. The intensity ratio between the D and G bands (I_D_/I_G_) is used to quantify the defect density in the graphene sheets [102]. The I_D_/I_G_ ratio for the GO was 0.93 (I_D_/I_G_ < 1), indicating the reduction in the sp^2^ domain of the GO surface due to the high contact potential difference between the GO and carboxyl functionalities on its surface [103]. The number of graphene layers increased as the intensity ratio increased, whereby the monolayer graphene had an intensity ratio of 0.28 [104]. These findings indicate that the GO synthesized using this method has the potential to exhibit bactericidal effects against bacteria cells [19,105,106].

### 3.2. Characterization of Functionalized PGO Nanofibers

**Figure 2:**
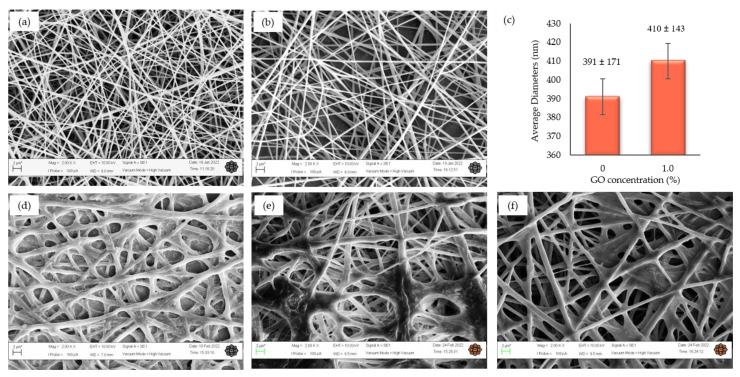
SEM images of (**a**) PCL/gelatin (PG); (**b**) PGO nanofibers; (**c**) average diameters of PG and PGO nanofibers; (**d**) PGO-M1; (**e**) PGO-M2; (**f**) PGO-M3 nanofibers at 2000 magnification (scale bar: 2 µm).

The resulting GO was loaded into the blended PG nanofibers. The surface morphologies of the PG and PGO (1% GO) nanofibers (Figure 2a,b) show the formation of homogeneous, uniform, and bead-free nanofibers, with average fiber diameters of 391 ± 171 nm and 410 ± 143 nm, respectively (Figure 2c). Several studies reported that the addition of GO causes an increase in solution conductivity, leading to an increase in fiber diameters [77,107,108]. Our study also showed similar results, whereby the addition of 1% *w/v* GO increased fiber diameters by 5%, signifying that the GO is well blended with the PG nanofibers. The increase in the fiber diameters may be due to the increase in the solution’s viscosity due to the interaction of GO with PCL/gelatin. In addition, the agglomeration of GO during the electrospinning process may also lead to the wide distribution of nanofibers of various sizes [109,110]. 

The surfaces of the nanofibers were functionalized with amino (-NH), carboxyl (-COO), and hydroxyl (-OH) groups of monomers by glow-discharge N plasma treatment to improve the wettability of the nanofibers’ surfaces for bacterial attachment [111]. The treatment was performed in a gaseous phase, whereby the plasma discharge, which ionized the gas with positive and negative charges, was introduced into the monomer vapor and sample surfaces. The plasma discharge caused fragmentations in the active precursor molecules of the monomers, leading to the deposition of monomers on the nanofibers’ surfaces [112,113]. Three monomers with different functional groups were used, i.e., diallylamine (M1), acrylic acid (M2), and *tert*-butyl acrylate (M3). The SEM images of the PGO nanofibers’ surfaces functionalized with the three monomers (Figure 2d,f) show the swelling and merging of the nanofibers after the treatment due to the grafting layers of the monomers on the surfaces of the nanofibers [113]. The morphology changes after the plasma treatment clearly indicate the successful deposition of monomers on the nanofibers’ surfaces [114]. 

**Figure 3:**
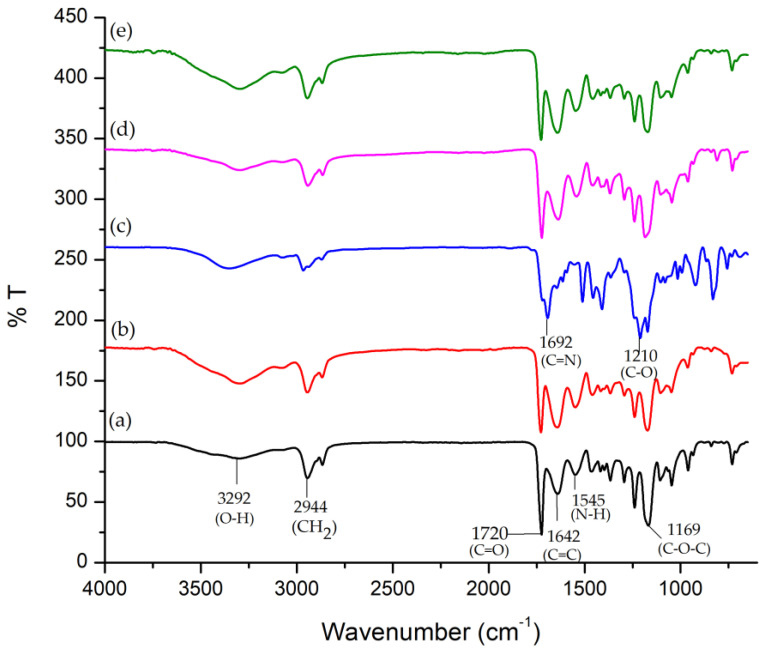
FTIR spectra of (**a**) PG; (**b**) PGO; (**c**) PGO-M1; (**d**) PGO-M2; and (**e**) PGO-M3 nanofibers.

The IR spectra of the functionalized nanofibers and the PG, PGO, PGO-M1, PGO-M2, and PGO-M3 nanofibers are shown in Figure 3. The PG ad PGO nanofibers had similar important bands observed at 3292 cm^−1^ (hydroxyl group), 2944 cm^−1^ (CH^2^ stretching), 1720 cm^−1^ (carbonyl stretching), 1642 cm^−1^ (C=C alkene), 1545 cm^−1^ (N-H stretching), and 1169 cm^−1^ (asymmetric C-O-C stretching), corresponding to the functional groups possessed by PCL, gelatin, and GO [77,115]. The intensities of the hydroxyl, alkene, and secondary amide peaks were higher in the PGO than in the PG nanofibers due to the interactions between the GO and the PG nanofibers, confirming the loading of GO into the nanofibers’ matrices [116].

**Figure 4:**
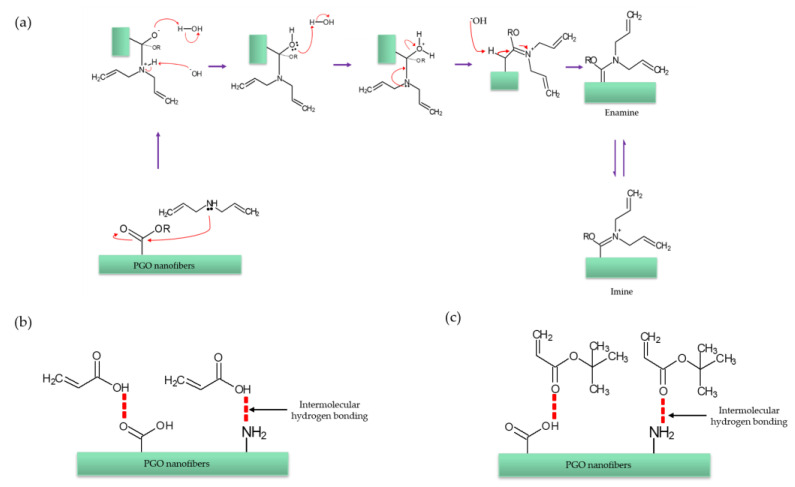
Interaction between monomers and nanofibers: (**a**) PGO-M1; (**b**) PGO-M2; (**c**) PGO-M3.

After the treatment with M1, two new bands were observed at 1692 cm^−1^ and 1210 cm^−1^, corresponding to imine (C=N) and ether (C-O) stretching, respectively. The results indicate that M1 was covalently bonded to the PGO nanofibers via the interaction between the secondary amine in diallylamine and the PGO, forming imine and enamine bonds, as illustrated by the reaction mechanism in Figure 4a. We propose that during the treatment, the electron pair of nitrogen in diallylamine reacts with the carbonyl group of the PGO nanofibers, forming a covalently bonded imine (C=N), which tautomerizes to enamine (C=C-N) [117,118,119]. For the PGO-M2 spectrum, the narrow peak at 1721 cm^−1^ (carbonyl group) and the observed 1641 cm^−1^ (C=C stretching) band confirm the presence of acrylic acid. The peak intensity at 1720 cm^−1^ and 1169 cm^−1^ increased slightly due to the hydrogen bonding interactions between the COOH and COC functionalities of the acrylic acid and PGO nanofibers (Figure 4b) [120]. The absorption bands at 1800 cm^−1^ (carbonyl group) and 1210 cm^−1^ (C-O stretching) in the PGO-M3 spectrum (Figure 3e) indicate the attachment of the *tert*-butyl acrylate monomer to the nanofibers. Similar bands were observed in the PGO-M3 spectrum due to the similar functional groups present in both the monomers and PGO nanofibers. Nonetheless, there was a marked increase in the intensity of the peak, especially at 3200 cm^−1^ (O-H stretching), 1640 cm^−1^ (C=C conjugated alkene), and 1545 cm^−1^ (N-H stretching) due to the strong hydrogen bonding interactions between the carbonyl group of the *tert*-butyl acrylate and the N-H bonds in the PGO nanofibers (Figure 4c) [121].

**Figure 5:**
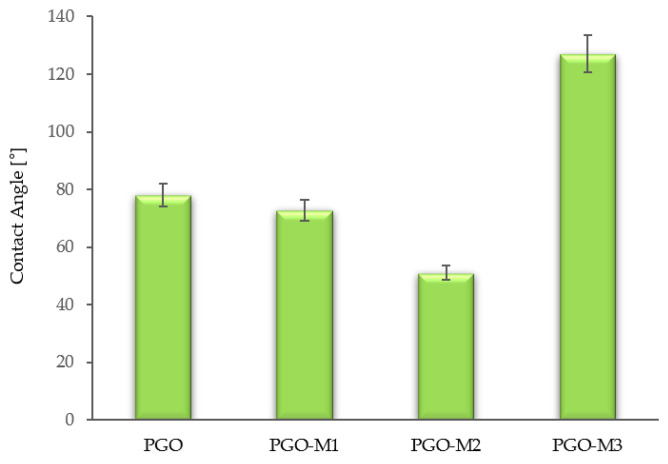
Contact angle [°] measurements of functionalized nanofibers.

The wettability of the functionalized nanofibers was analyzed by water contact angle measurements (Figure 5). The water contact angle of the PGO nanofibers was 78 ± 1.4°, indicating high wettability due to the incorporation of gelatin and graphene oxide. The improved hydrophilicity of the nanofibers was mainly due to the incorporation of gelatin and graphene oxide containing -COOH and -OH groups, rendering the PGO nanofibers with high water affinity compared to pure PCL, which is hydrophobic in nature, with a water contact angle of 118 ± 6° [122,123]. The water contact angle of PGO-M1 was 72.6 ± 1.3°, which contained the polar and hydrophilic N-H group of diallylamine [124]. The PGO-M2 nanofibers functionalized with acrylic acid gave a water contact angle of 51.05 ± 1.9°. The -COOH group of acrylic acid contributed to its miscibility in water, giving the lowest wettability among the three samples [125]. However, the PGO nanofibers functionalized with M3 (*tert*-butyl acrylate) displayed a water contact angle measurement of 127.03 ± 17.5° due to the hydrophobic surface provided by the *tert*-butyl acrylate [126]. 

**Figure 6:**
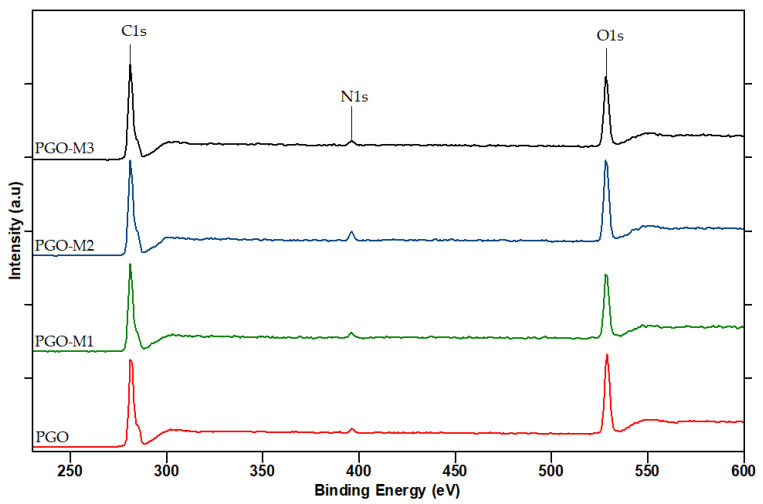
XPS survey scans of PGO, PGO-M1, PGO-M2, and PGO-M3 nanofibers.

An XPS survey analysis was performed to further confirm the deposition and chemical bonding between the PGO and the monomers (Figure 6). The nanofibers’ spectra exhibited C1s, O1s, and N1s peaks at 285, 531, and 398 eV, respectively [127,128]. The atomic concentrations and ratios obtained from the deconvolution of the survey analysis are summarized in Table 1. It was possible to recognize six component peaks retained in the curve fitting at 284.5 eV (C-C), 285.9 eV (C-O), 288.5 eV (C-N), 531.1 eV (C=O), 532.4 eV (O-H), and 398.0 eV (N-H). Only slight changes were detected in the atomic concentration of the PGO nanofibers after the plasma treatment. The M1 and M3 coatings of PGO showed a significant increase in the C1s concentration and a decrease in the O1s concentration. The increase in carbon atoms in the nanofibers’ backbones may reduce their hydrophilicity (Figure 5). The plasma treatment of M1 resulted in nanofibers rich in N with an N/C ratio of 0.36 compared to PGO nanofibers, confirming the formation of imine and enamine, as illustrated previously (Figure 4). Meanwhile, the N/C concentration in the M3 coating was reduced slightly in the PGO nanofibers, indicating the deposition of *tert*-butyl acrylate on the nanofibers. Hence, the higher concentration of the polar N atom in PGO-M1 makes it slightly more hydrophilic compared to the PGO-M3 nanofibers, which agrees with the contact angle measurement (Figure 5).

In contrast, the C1s concentration of the M2 coating showed a retention of carbon on the surfaces of the PGO nanofibers, whereas the O1s concentration increased and the N1s concentration decreased on the PGO-M2 nanofibers, thus confirming the oxygen binding on the fibers’ surfaces [110]. It is worth noting that the higher concentration of oxygen further increased the hydrophilicity of the nanofibers. Overall, the results of the XPS survey are in agreement with the results of the wettability analysis, confirming that a copious amount of monomers was deposited on the PGO nanofibers’ surfaces [79,127,129].

**Figure 7:**
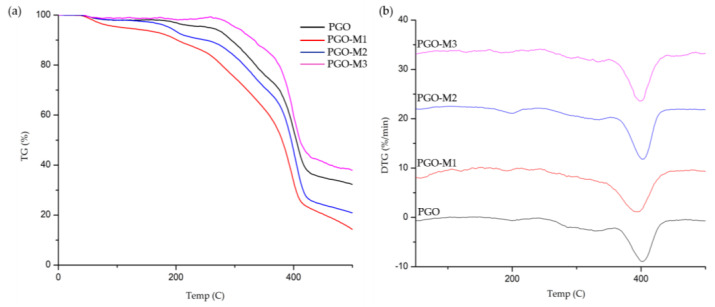
(**a**) TGA and (**b**) DTG of functionalized nanofibers.

The thermal stability of the nanofibers was examined using TGA and DTG analyses (Figure 7). The thermal degradation of the PGO and PGO-M2 nanofibers took place in three ranges: 30–170, 180–380, and 400–500 °C. Meanwhile, the thermal degradation of the PGO-M1 nanofibers occurred at 30–60, 90–360, and 400–500 °C, and for the PGO-M3 nanofibers, at 30–230, 270–370, and 400–500 °C. After 500 °C, the total weight losses for PGO, PGO-M1, PGO-M2, and PGO-M3 were 67.97, 86.1, 79.32, and 62.25%, respectively. The initial weight loss of the nanofibers was due to the loss of volatile components such as moisture through evaporation [130,131,132]. Significant weight losses for PGO, PGO-M1, PGO-M2, and PGO-M3 occurred at 180, 100, 180, and 270 °C, respectively. The thermal stabilities of PGO-M1 and PGO-M2 were reduced compared to the PGO nanofibers, which may be related to the morphological changes in the PGO when treated with M1 and M2, as shown in the SEM images (Figure 2) [131]. The treatment further increased the surface area of the PGO nanofibers, thus providing a greater area for water adsorption and reducing its thermal stability [132]. In contrast, PGO-M3 showed an increase in thermal stability as it started to degrade at higher temperatures, with a smaller weight loss (%) compared to the PGO nanofibers. The strong hydrogen bonding interactions between the PGO nanofibers and M3 may have contributed to the results, as a higher temperature was required to break the bonds [133,134]. Our results showed that PGO-M1 provided the best thermal degradation and stability, followed by PGO-M2, PGO, and PGO-M3.

**Figure 8:**
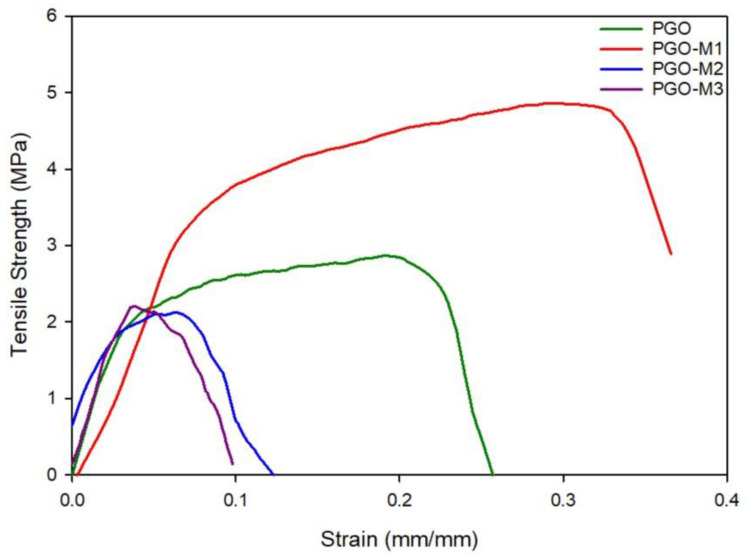
Stress–strain curve of the functionalized nanofibers.

The tensile properties of the functionalized PGO nanofibers were measured to determine their durability and elasticity. A tensile stress–strain curve of the functionalized nanofibers was obtained, and the tensile strength, strain break, as well as Young’s modulus, were calculated from the curve [135,136]. Figure 8 shows the elastic deformation of the functionalized nanofibers. As the deformation increased, the sample entered the yield stage or elastic limit, where the material was deformed and beyond recovery [137]. The tensile strength is the maximum value of stress (y-axis), whereas the strain break is the maximum point of strain (x-axis), obtained from the curve. The Young’s modulus of the functionalized nanofibers was obtained from the initial slope or gradient of the stress–strain curve. The results are summarized in Table 2.

The PGO nanofibers recorded a strength, strain break, and Young’s modulus of 2.87 ± 0.81 MPa, 26.22 ± 7.31%, and 66.96 ± 6.42 MPa, respectively. The value of Young’s modulus indicates the high stiffness of the samples, which is inversely proportional to the elasticity. The blending of GO further improved the tensile strength of the PG nanofibers due to the hydrogen bonding interactions between GO and the nanofibers [22,135,136,138]. PGO-M1 showed the highest tensile strength and strain break compared to the PGO nanofibers. The covalently bonded M1 may contribute to the improved elasticity of the nanofibers. The high surface area of the PGO-M1 nanofibers provides support against the stress applied on the nanofibers’ surfaces, thus improving their mechanical properties [139]. In contrast, the PGO-M2 and PGO-M3 nanofibers showed reductions in tensile strength and elongations of the strain break. This may be due to the acidic properties of the acrylic acid (M2) and *tert*-butyl acrylate (M3), which can affect the nanofibers’ elasticity [140,141]. 

### 3.3. Antimicrobial Response

#### 3.3.1. Plate-Counting Assay

The plate-counting assay was performed to evaluate the antimicrobial efficiency of the nanofibers against *S. aureus* and *E. coli* before and after the plasma surface treatment, with the PG nanofibers as the control. The results are represented in Table 3. The PG nanofibers did not exhibit antimicrobial activity against both bacteria strains. After incubation with the PGO nanofibers, there was no *E. coli* colony growth observed on the plate, whereas a small amount of S. aureus colonies (9.0 × 10^–6^ CFU/mL) were detected on the agar plate. Previous studies reported that the sharp edges of GO in nanofibers could induce membrane stress and destroy the phospholipid bilayers of bacteria, leading to physical damage to the bacterial membrane integrity [142,143,144]. Nevertheless, the PGO nanofibers were more effective in killing the Gram-negative bacteria *E. coli* compared to the Gram-positive bacteria *S. aureus*. This may be due to the rigid cell walls of Gram-positive bacteria, which prevent GO from penetrating the bacterial membrane [145,146]. 

The nanofibers retained their antibacterial activity after being treated with M1 and M2, with a slightly increased effectiveness against *S. aureus*. However, the PGO-M3 antibacterial activity was reduced, with the highest colony count compared to PGO, PGO-M1, and PGO-M2. The presence of a positively charged amino group in M1 may have induced toxicity to the bacteria cells [78,147]. On the other hand, although the acrylic acid, M2, was negatively charged, the antibacterial effect of the nanofibers was attributed to the acidic nature of M2, which reduced the cytoplasmic pH level of bacteria cells [148] and disrupted the ion-exchange mechanism of the cell membrane, resulting in cell lysis [149]. As for M3, the acrylate group was reported to have a significantly high bacterial attachment due to its polarity and linear structure [150]. However, the coating of the PGO-M3 nanofibers reduced the antibacterial activity of GO, which may have been due to the strong hydrogen bonding interactions between M3 and the PGO nanofibers, as shown in Figure 3, thus limiting the direct interaction of GO and bacteria, including the ability to kill them [150,151,152,153].

#### 3.3.2. Bacterial Attachment on Functionalized Nanofibers

**Figure 9:**
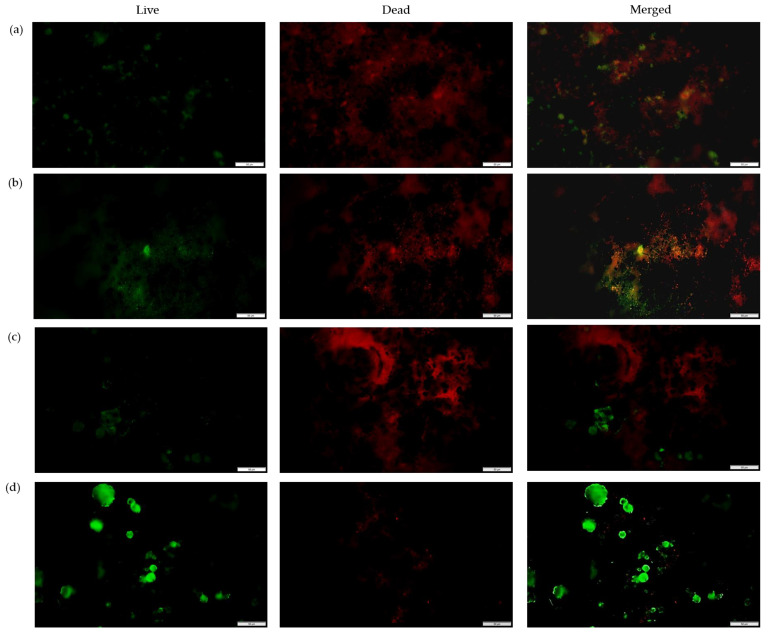
Fluorescence microscopy images of *S. aureus* attachment on (**a**) PGO; (**b**) PGO-M1; (**c**) PGO-M2; and (**d**) PGO-M3 nanofibers at 40× magnification (scale bar: 50 µm).

**Figure 10:**
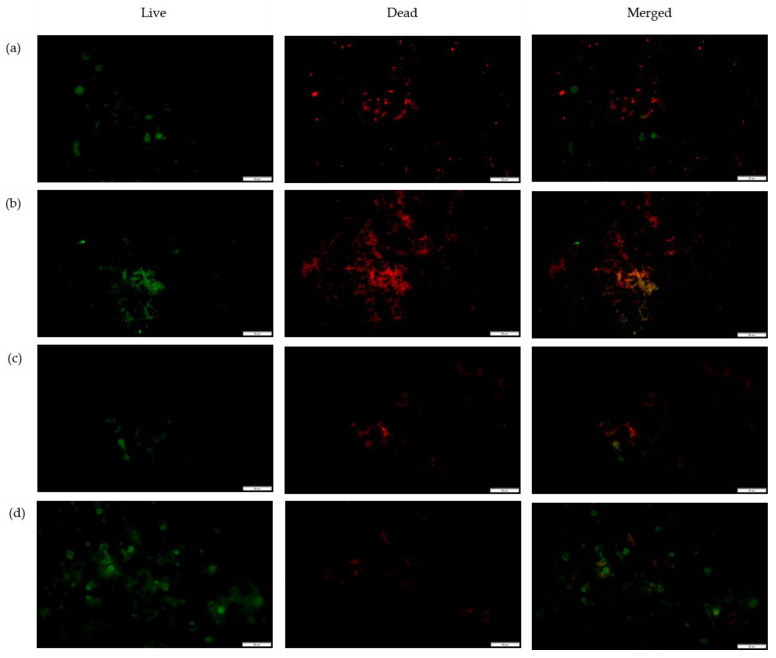
Fluorescence microscopy images of *E. coli* attachment on (**a**) PGO; (**b**) PGO-M1; (**c**) PGO-M2; and (**d**) PGO-M3 nanofibers at 40× magnification (scale bar: 50 µm).

**Figure 11:**
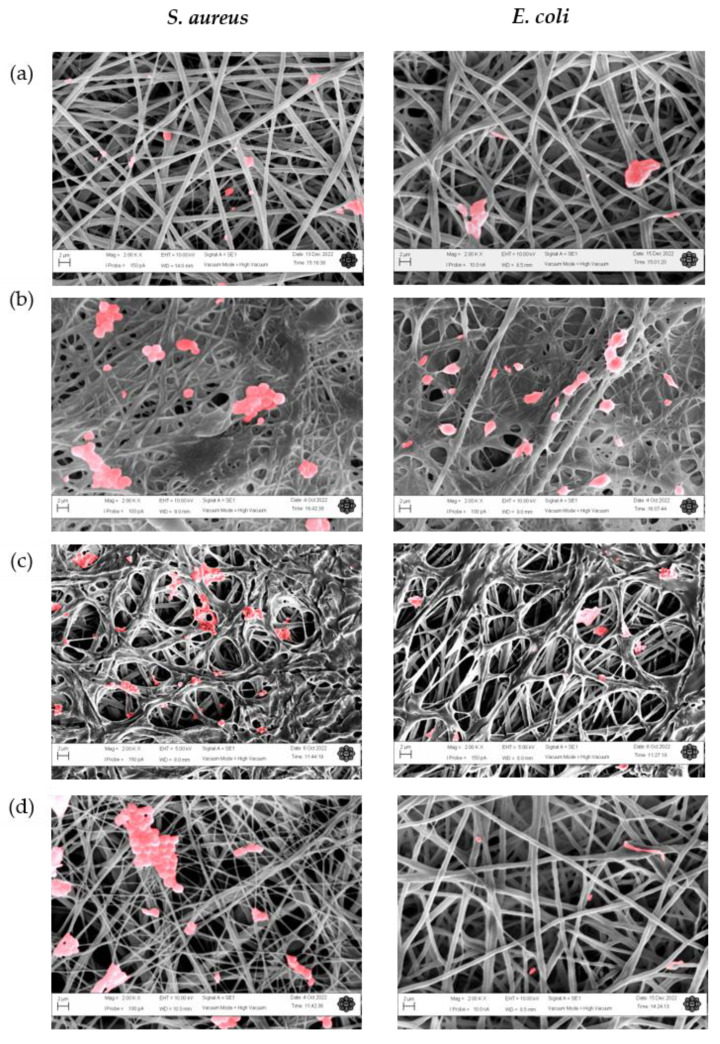
SEM images of bacteria attachment on (**a**) PGO; (**b**) PGO-M1; (**c**) PGO-M2; and (**d**) PGO-M3 nanofibers at 2000 magnification. Red indicates bacteria colonies (Scale bar: 2 µm).

To gain insight into the influence of the monomers on bacterial attachment, the nanofibers’ surfaces were investigated after incubation with *S. aureus* (Figure 9) and *E. coli* (Figure 10) via fluorescence microscopy and SEM (Figure 11), with the PGO nanofibers as the control. Our study found that the attachment of *S. aureus* to the nanofibers’ surfaces (Figure 9) was higher compared to *E. coli.* This was due to the size of the nanofibers, which influenced the attachment of the bacteria. Previously, Abrigo et al. reported that bacterial adhesion and attachment to fibers’ surfaces are influenced by the fibers’ diameters, whereby the bacteria tend to adhere to fibers that are closer to their size [154]. *S. aureus* has an average size of 0.5–1.5 µm, whereas *E. coli* has an average size of 1.0–3.0 µm. Since the functionalized nanofibers’ sizes were below 1000 nm, the attachment of *S. aureus* was higher compared to *E. coli*. The green fluorescence of the nanofibers’ surfaces indicated a live bacteria cell, whereas red fluorescence indicated a dead cell. For the PGO nanofibers, there was a small amount of *E. coli* attachment observed compared to *S. aureus*. The slightly hydrophobic PGO nanofibers may have been more attracted to the hydrophobic *S. aureus* than the *E. coli* [58,155]. Large clusters of live *S. aureus* cells were found on the nanofibers’ surfaces compared to *E. coli* cells, indicating that graphene oxide was more effective in killing *E. coli* than *S. aureus*, as discussed previously. 

The positively charged PGO-M1 strongly attracted the negatively charged *S. aureus*, thus promoting attachment to the nanofibers’ surfaces [78,156]. A small number of live *S. aureus* colonies were attached to PGO-M1 compared to PGO nanofibers, indicating that the diallylamine coating improved the antimicrobial efficiency of PGO nanofibers against *S. aureus.* On the other hand, the highest number of dead *E. coli* colonies was found attached to the PGO-M1 nanofibers. The increase in surface wettability of the PGO-M1 nanofibers promoted the adhesion of *E. coli* colonies to their surfaces compared to the other samples. The distorted rod-shaped morphology of *E. coli* indicates that the functionalized nanofibers changed the bacterial conformation, leading to cell death (Figure 11b). 

The attachment of *S. aureus* and *E. coli* was decreased in the PGO-M2 nanofibers compared to the PGO nanofibers, which may be due to the negatively charged acrylic acid under a neutral pH, which repelled the negatively charged bacteria cells [73]. The dead bacteria cells were attached to the PGO-M2 surface due to the acidic property of M2, which triggered the bacteria attachment. Similarly, Wu et al. (2018) found that a large number of dead *E. coli* and *S. aureus* colonies were attached to multi-layered dopamine–polyacrylic acid and chitosan quaternary ammonium salt polymer grafting when the pH level of the surface decreased, indicating that bacteria attachment can be triggered in an acidic environment [157]. For the PGO-M3 nanofibers, a small number of *E. coli* and a large number of *S. aureus* colonies were observed on the nanofibers’ surfaces. This may be due to the hydrophobicity of M3 attracting the hydrophobic *S. aureus* colonies better than the *E. coli* colonies [158]. As we can see in Figure 11d,e, *S. aureus* was attracted and attached to the smaller-sized fibers compared to *E. coli* [159]. Although it had a better attachment to the nanofibers, we can see the large clusters of live bacteria cells that were attached to the nanofibers (Figure 9d and Figure 10d), indicating that the contact-killing action of the PGO-M3 nanofibers toward the killing bacteria was low. In addition, there were no changes in the *S. aureus* and *E. coli* morphologies (Figure 11d), indicating that the live bacteria cells were attached to the sample. These findings correlate with the results from the previous plate-counting assay (see Table 3). Based on these findings, the attachment of bacteria can be influenced by several factors; thus, further studies to investigate the influence of these factors are imperative for the proper design of effective antimicrobial functional materials.

### 3.4. In Vitro Studies

**Figure 12:**
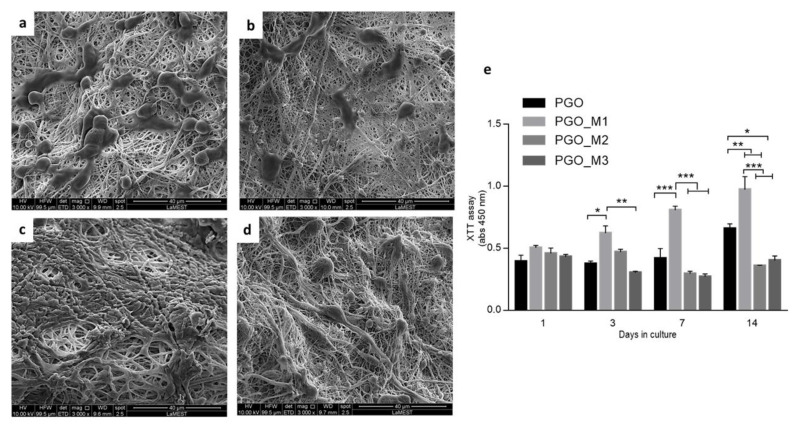
SEM images of L929 cells onto PGO (**a**), PGO-M1 (**b**), PGO-M2 (**c**), and PGO-M3 (**d**) nanofibers. Scale bar: 40 μm. (**e**) XTT assay for biocompatibility of nanofibers at 1, 3, 7, and 14 days. Results are presented as the average ± standard error of the mean. * Represents statistical significance (* *p* < 0.05, ** *p* < 0.01, *** *p* < 0.001).

The interaction of the cells and nanofibers after 48 h is shown in Figure 12. In the PGO fibers, clusters of cells and slightly flattened morphology can be observed (Figure 11a). The nanofibers with diallylamine monomer (M1) cells presented a more elongated morphology and marked cytoplasmatic prolongations (Figure 12b), which may be related to the presence of the amine group that allowed the electrostatic interaction with the cells [160], unlike PGO-M2 (Figure 12c). On the other hand, the cells were able to interact with PGO-M3 nanofibers due to the polarity of the *tert*-butyl acrylate favoring cell adhesion (Figure 12d).

The biocompatibility of biomaterials refers to the ability of the material to avoid causing a cytotoxic response. Moreover, biomaterials should provide a favorable microenvironment involving physical and chemical properties [161]. PCL and gelatin nanofibers have been widely studied showing good biocompatibility due to the improvement of hydrophilicity and the affinity of the cell to gelatin, favoring cell adhesion, proliferation, and differentiation [162,163,164,165,166]. The current results show that the presence of GO in the nanofibers caused a cytotoxic effect by decreasing the metabolic activity based on the XTT assay, despite the presence of gelatin (Figure 12e). The chemistry and surface charge of materials influence biocompatibility and cellular processes. In particular, GO can interact with the lipid tail of phospholipids in the cell membrane, removing cholesterol molecules and leading to membrane damage [167]. In this work, PGO nanofibers were further functionalized and analyzed in terms of biocompatibility. The PGO nanofibers functionalized with a positively charged group (M1) reduced the hydrophobicity and allowed cell activity over time. On the other hand, a further increase in surface hydrophilicity in the case of the acrylic acid treatment (PGO-M2) generated an excess of negative charges onto the surface that promoted electrostatic repulsion, leading to cell damage. Lastly, the polar tert-butyl acrylate (M3), in combination with the negative charge of GO, decreased the metabolic activity of cells. 

## 4. Conclusions

GO-PCL/gelatin electrospun nanofibers were successfully functionalized by grafting diallylamine (M1), acrylic acid (M2), or *tert*-butyl acrylate (M3) via plasma treatment to improve the surface properties of antibacterial nanofibers. The fabrication of the functionalized nanofibers was confirmed by SEM, FTIR, XPS, wettability measurements, TGA studies, and tensile strength measurements. The functional groups possessed by each monomer affected the surface wettability, with PGO-M2 as the most hydrophilic and PGO-M3 as the most hydrophobic. The hydrophilic behavior of the surfaces, strictly related to the chemical functionalization on the nanofibers, significantly influenced in vitro cell and antibacterial properties: higher *S. aureus* colony growth was observed in PGO-M3 than in PGO-M1 and PGO-M2. Meanwhile, L929 tended to better adhere and proliferate to PGO-M1. Accordingly, it was found that antimicrobial activity against *E. coli* bacteria was the highest in PGO-M1, followed by PGO-M2 and PGO-M3, thus confirming the selective interaction of functional groups and different bacteria populations. 

Further studies could create a better understanding of the mechanisms and efficiency of bactericidal interaction. This may pave the way for the design of innovative non-antibiotic treatments that are suitable for efficiently reducing the formation of biofilms, leading to the development of new solutions for current health and environmental problems.

## Figures and Tables

**Table 1:** Atomic concentrations (%) of functionalized nanofibers based on deconvolution of XPS spectra.

Sample	Atomic Concentration (%)				
	C1s	O1s	N1s	O/C	N/C
PGO	37.61	49.85	12.53	1.30	0.33
PGO-M1	41.58	43.26	15.16	1.04	0.36
PGO-M2	37.79	53.90	8.30	1.43	0.22
PGO-M3	43.42	44.99	11.59	1.04	0.27

**Table 2:** Tensile properties of the functionalized PGO nanofibers.

Nanofibers	Tensile Strength (MPa)	Strain Break (%)	Young’s Modulus (MPa)
PGO	2.87 ± 0.13 *	26.22 ± 2.13 *	66.96 ± 6.42 *
PGO-M1	4.86 ± 1.57 *	36.60 ± 8.62 *	46.91 ± 1.04 *
PGO-M2	2.12 ± 0.63 *	12.60 ± 1.66 *	65.46 ± 9.32 *
PGO-M3	2.21 ± 1.15 *	9.80 ± 9.89 *	74.94 ± 5.05 *

* *p* value is < 0.001, the data are considered statistically significant (*p* < 0.05).

**Table 3:** Antimicrobial activity of nanofibers against *S. aureus* and *E. coli* (average CFU/mL).

Nanofibers	*E. coli*	*S. aureus*
PG (control)	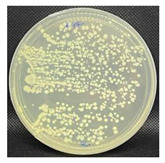 Uncountable	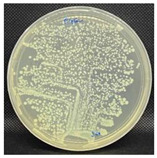 Uncountable
PGO	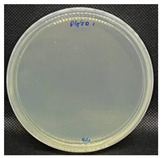 No growth	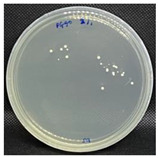 9.0 × 10^−6^ CFU/mL
PGO-M1	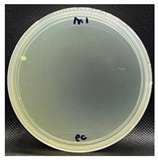 5.0 × 10^−7^ CFU/mL	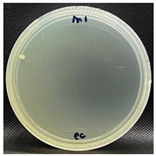 5.0 × 10^−7^ CFU/mL
PGO-M2	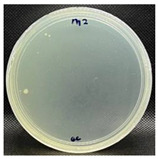 2.5 × 10^−6^ CFU/mL	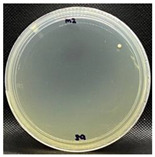 1.0 × 10^−6^ CFU/mL
PGO-M3	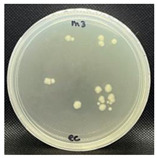 8.5 × 10^−6^ CFU/mL	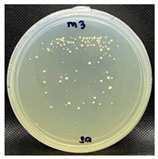 5.5 × 10^−5^ CFU/mL

## Data Availability

Not applicable.

## References

[1] (2019). US Department of Health and Human Services, Centres for Disease Control and Prevention: Atlanta, GA, USA. https://en.wikipedia.org/.

[2] Elsayed A.A., Darwish S.F., Zewail M.B., Mohammed M., Saeed H., Rabea H. (2021). Antibiotic misuse and compliance with infection control measures during COVID-19 pandemic in community pharmacies in Egypt. Int. J. Clin. Pract..

[3] Mallah N., Orsini N., Figueiras A., Takkouche B. (2022). Income level and antibiotic misuse: A systematic review and dose–response meta-analysis. Eur. J. Health Econ..

[4] Shao Y., Wang Y., Yuan Y., Xie Y. (2021). A systematic review on antibiotics misuse in livestock and aquaculture and regulation implications in China. Sci. Total. Environ..

[5] Diamant M., Baruch S., Kassem E., Muhsen K., Samet D., Leshno M. (2021). A game theoretic approach reveals that discreetizing clinical information can reduce antibiotic misuse. Nat. Commun..

[6] Serwecińska L. (2020). Antimicrobials and Antibiotic-Resistant Bacteria: A Risk to the Environment and to Public Health. Water.

[7] Mancuso G., Midiri A., Gerace E., Biondo C. (2021). Bacterial Antibiotic Resistance: The Most Critical Pathogens. Pathogens.

[8] Bowler P.G. (2018). Antibiotic resistance and biofilm tolerance: A combined threat in the treatment of chronic infections. J. Wound Care.

[9] Abdel Maksoud M.I.A., El-Sayyad G.S., El-Bastawisy H.S., Fathy R.M. (2021). Antibacterial and antibiofilm activities of silver-decorated zinc ferrite nanoparticles synthesized by a gamma irradiation-coupled sol–gel method against some pathogenic bacteria from medical operating room surfaces. RSC Adv..

[10] Ansari M.A., Albetran H.M., Alheshibri M.H., Timoumi A., Algarou N.A., Akhtar S., Slimani Y., Almessiere M.A., AlAhmari F.S., Baykal A. (2020). Synthesis of Electrospun TiO_2_ Nanofibers and Characterization of Their Antibacterial and Antibiofilm Potential against Gram-Positive and Gram-Negative Bacteria. Antibiotics.

[11] Grant J.J., Pillai S.C., Hehir S., McAfee M., Breen A. (2021). Biomedical Applications of Electrospun Graphene Oxide. ACS Biomater. Sci. Eng..

[12] El-Naggar M.E., Elmushyakhi A., Al-Sehemi A.G., Kalam A., Algarni H., Salem S.R., Taleb M.A. (2022). Biomedical domains of the as-prepared nanocomposite based on hydroxyapatite, bismuth trioxide and graphene oxide. J. Mater. Res. Technol..

[13] Rivera-Briso A.L., Aachmann F.L., Moreno-Manzano V., Serrano-Aroca A. (2020). Graphene oxide nanosheets versus carbon nanofibers: Enhancement of physical and biological properties of poly(3-hydroxybutyrate-co-3-hydroxyvalerate) films for biomedical applications. Int. J. Biol. Macromol..

[14] Sun M., Li J. (2018). Graphene oxide membranes: Functional structures, preparation and environmental applications. Nano Today.

[15] Yu W., Sisi L., Haiyan Y., Jie L. (2020). Progress in the functional modification of graphene/graphene oxide: A review. RSC Adv..

[16] Brisebois P.P., Siaj M. (2020). Harvesting graphene oxide—Years 1859 to 2019: A review of its structure, synthesis, properties and exfoliation. J Mater Chem C.

[17] Yu C.-H., Chen G.-Y., Xia M.-Y., Xie Y., Chi Y.-Q., He Z.-Y., Zhang C.-L., Zhang T., Chen Q.-M., Peng Q. (2020). Understanding the sheet size-antibacterial activity relationship of graphene oxide and the nano-bio interaction-based physical mechanisms. Colloids Surf. B Biointerfaces.

[18] Mokkapati V.R.S.S., Pandit S., Kim J., Martensson A., Lovmar M., Westerlund F., Mijakovic I. (2018). Bacterial response to graphene oxide and reduced graphene oxide integrated in agar plates. R. Soc. Open Sci..

[19] Qiu J., Liu L., Zhu H., Liu X. (2018). Combination types between graphene oxide and substrate affect the antibacterial activity. Bioact. Mater..

[20] Lu C.-J., Jiang X.-F., Junaid M., Ma Y.-B., Jia P.-P., Wang H.-B., Pei D.-S. (2017). Graphene oxide nanosheets induce DNA damage and activate the base excision repair (BER) signaling pathway both in vitro and in vivo. Chemosphere.

[21] Gurunathan S., Iqbal M.A., Qasim M., Park C.H., Yoo H., Hwang J.H., Uhm S.J., Song H., Park C., Do J.T. (2019). Evaluation of Graphene Oxide Induced Cellular Toxicity and Transcriptome Analysis in Human Embryonic Kidney Cells. Nanomaterials.

[22] Heidari M., Bahrami H., Ranjbar-Mohammadi M. (2017). Fabrication, optimization and characterization of electrospun poly(caprolactone)/gelatin/graphene nanofibrous mats. Mater. Sci. Eng. C.

[23] Contreras-Cáceres R., Cabeza L., Perazzoli G., Díaz A., López-Romero J.M., Melguizo C., Prados J. (2019). Electrospun Nanofibers: Recent Applications in Drug Delivery and Cancer Therapy. Nanomaterials.

[24] Baykara T., Taylan G. (2021). Coaxial electrospinning of PVA/Nigella seed oil nanofibers: Processing and morphological characterization. Mater. Sci. Eng. B.

[25] Sankaran S., Deshmukh K., Basheer A.M., Khadheer P.S.K., Sadasivuni K., Ponnamma D., Rajan M., Ahmed B., Al-Maadeed M. (2019). Electrospun Polymeric Nanofibers: Fundamental aspects of electrospinning processes, optimization of electrospinning parameters, properties, and applications. Polymer Nanocomposites in Biomedical Engineering.

[26] Thangavel K., Roshini T., Balaprakash V., Gowrisankar P., Sudha S., Mohan M. (2019). Structural, morphological and antibacterial properties of ZnO nanofibers fabricated by electrospinning technique. Mater. Today Proc..

[27] Kegere J., Ouf A., Siam R., Mamdouh W. (2019). Fabrication of Poly(vinyl alcohol)/Chitosan/*Bidens pilosa* Composite Electrospun Nanofibers with Enhanced Antibacterial Activities. ACS Omega.

[28] Gupta A., Mumtaz S., Li C.-H., Hussain I., Rotello V.M. (2019). Combatting antibiotic-resistant bacteria using nanomaterials. Chem. Soc. Rev..

[29] Borjihan Q., Dong A. (2020). Design of nanoengineered antibacterial polymers for biomedical applications. Biomater. Sci..

[30] Yang J., Wang K., Yu D.-G., Yang Y., Bligh S.W.A., Williams G.R. (2020). Electrospun Janus nanofibers loaded with a drug and inorganic nanoparticles as an effective antibacterial wound dressing. Mater. Sci. Eng. C.

[31] Memic A., Abudula T., Mohammed H.S., Joshi Navare K., Colombani T., Bencherif S.A. (2019). Latest Progress in Electrospun Nanofibers for Wound Healing Applications. ACS Appl. Bio Mater..

[32] Barhoum A., Pal K., Rahier H., Uludag H., Kim I.S., Bechelany M. (2019). Nanofibers as new-generation materials: From spinning and nano-spinning fabrication techniques to emerging applications. Appl. Mater. Today.

[33] Kadavil H., Zagho M., Elzatahry A., Altahtamouni T. (2019). Sputtering of Electrospun Polymer-Based Nanofibers for Biomedical Applications: A Perspective. Nanomaterials.

[34] Brianezi S.F.S., Castro K.C., Piazza R.D., Melo M.S.F., Pereira R.M., Marques R.F.C., Campos M.G.N. (2018). Preparation and characterization of Chitosan/MPEG-PCL blended membranes for wound dressing and controlled gentamicin release. Mater. Res..

[35] Jafari A., Amirsadeghi A., Hassanajili S., Azarpira N. (2020). Bioactive antibacterial bilayer PCL/gelatin nanofibrous scaffold promotes full-thickness wound healing. Int. J. Pharm..

[36] Fasolino I., Guarino V., Cirillo V., Ambrosio L. (2017). 5-Azacytidine-mediated hMSC behavior on electrospun scaffolds for skeletal muscle regeneration. J. Biomed. Mater. Res. Part A.

[37] Ehrmann A. (2021). Non-Toxic Crosslinking of Electrospun Gelatin Nanofibers for Tissue Engineering and Biomedicine—A Review. Polymers.

[38] Xie X., Li D., Chen Y., Shen Y., Yu F., Wang W. (2021). Conjugate electrospun 3D gelatin nanofiber sponge for rapid hemostasis. Adv Healthc Mater.

[39] Vineis C., Maya I.C., Mowafi S., Varesano A., Ramírez D.S., Taleb M.A., Tonetti C., Guarino V., El-Sayed H. (2021). Synergistic effect of sericin and keratin in gelatin based nanofibers for in vitro applications. Int. J. Biol. Macromol..

[40] Abdul Khodir W.K.W., Abdul Razak A.H., Ng M.H., Guarino V., Susanti D. (2018). Encapsulation and Characterization of Gentamicin Sulfate in the Collagen Added Electrospun Nanofibers for Skin Regeneration. J. Funct. Biomater..

[41] García-Valderrama E.J., Mamidi N., Antunes-Ricardo M., Gutiérrez-Uribe J.A., Del Angel-Sanchez K., Elías-Zúñiga A. (2022). Engineering and Evaluation of Forcespun Gelatin Nanofibers as an Isorhamnetin Glycosides Delivery System. Pharmaceutics.

[42] Li T., Sun M., Wu S. (2022). State-of-the-Art Review of Electrospun Gelatin-Based Nanofiber Dressings for Wound Healing Applications. Nanomaterials.

[43] Lin L., Gu Y., Cui H. (2018). Novel electrospun gelatin-glycerin-ε-Poly-lysine nanofibers for controlling Listeria monocytogenes on beef. Food Packag. Shelf Life.

[44] Atashgahi M., Ghaemi B., Valizadeh A., Moshiri A., Nekoofar M.H., Amani A. (2021). Epinephrine-entrapped chitosan nanoparticles covered by gelatin nanofibers: A bi-layer nano-biomaterial for rapid hemostasis. Int. J. Pharm..

[45] Gounani Z., Pourianejad S., Asadollahi M.A., Meyer R.L., Rosenholm J.M., Arpanaei A. (2020). Polycaprolactone-gelatin nanofibers incorporated with dual antibiotic-loaded carboxyl-modified silica nanoparticles. J. Mater. Sci..

[46] Guerreiro S.F.C., Valente J.F.A., Dias J.R., Alves N. (2021). Box-Behnken Design a Key Tool to Achieve Optimized PCL/Gelatin Electrospun Mesh. Macromol. Mater. Eng..

[47] Kalantary S., Jahani A., Pourbabaki R., Beigzadeh Z. (2019). Application of ANN modeling techniques in the prediction of the diameter of PCL/gelatin nanofibers in environmental and medical studies. RSC Adv..

[48] Li Y., Dong T., Li Z., Ni S., Zhou F., Alimi O.A., Chen S., Duan B., Kuss M., Wu S. (2022). Review of advances in electrospinning-based strategies for spinal cord regeneration. Mater. Today Chem..

[49] Sun M., Chen S., Ling P., Ma J., Wu S. (2022). Electrospun Methacrylated Gelatin/Poly(L-Lactic Acid) Nanofibrous Hydrogel Scaffolds for Potential Wound Dressing Application. Nanomaterials.

[50] Zhou Y., Liu Y., Zhang M., Feng Z., Yu D.-G., Wang K. (2022). Electrospun Nanofiber Membranes for Air Filtration: A Review. Nanomaterials.

[51] Avcu E., Bastan F.E., Guney M., Avcu Y.Y., Rehman M.A.U., Boccaccini A.R. (2022). Biodegradable Polymer Matrix Composites Containing Graphene-Related Materials for Antibacterial Applications: A Critical Review. Acta Biomater..

[52] Miguel S.P., Figueira D.R., Simoes D., Ribeiro M.P., Coutinho P., Ferreira P. (2018). Electrospun polymeric nanofibers as wound dressings: A review. Colloids Surf. B Biointerfaces.

[53] Kurtz I.S., Schiffman J.D. (2018). Current and Emerging Approaches to Engineer Antibacterial and Antifouling Electrospun Nanofibers. Materials.

[54] Lencova S., Svarcova V., Stiborova H., Demnerova K., Jencova V., Hozdova K., Zdenkova K. (2021). Bacterial Biofilms on Polyamide Nanofibers: Factors Influencing Biofilm Formation and Evaluation. ACS Appl. Mater. Interfaces.

[55] De Cesare F., Di Mattia E., Zussman E., Macagnano A. (2019). A study on the dependence of bacteria adhesion on the polymer nanofibre diameter. Environ. Sci. Nano.

[56] Gördegir M., Oz S., Yezer I., Buhur M., Unal B., Demirkol D.O. (2019). Cells-on-nanofibers: Effect of polyethyleneimine on hydrophobicity of poly-Ɛ-caprolacton electrospun nanofibers and immobilization of bacteria. Enzym. Microb. Technol..

[57] Zupancic S., Skrlec K., Kocbek P., Berlec A. (2019). Effects of electrospinning on the viability of ten species of lactic acid bacteria in poly(ethylene oxide) nanofibers. Pharmaceutics.

[58] Sarker A., Tran N., Rifai A., Brandt M., Tran P., Leary M., Fox K., Williams R. (2019). Rational design of additively manufactured Ti6Al4V implants to control Staphylococcus aureus biofilm formation. Materialia.

[59] Yusuf Y., Ghazali M.J., Otsuka Y., Ohnuma K., Morakul S., Nakamura S. (2020). Antibacterial properties of laser surface-textured TiO2/ZnO ceramic coatings. Ceram Int.

[60] Lu A., Gao Y., Jin T., Luo X., Zeng Q., Shang Z. (2020). Effects of surface roughness and texture on the bacterial adhesion on the bearing surface of bio-ceramic joint implants: An in vitro study. Ceram. Int..

[61] Khosravimelal S., Chizari M., Farhadihosseinabadi B., Moghaddam M.M., Gholipourmalekabadi M. (2021). Fabrication and characterization of an antibacterial chitosan/silk fibroin electrospun nanofiber loaded with a cationic peptide for wound-dressing application. J. Mater. Sci. Mater. Med..

[62] Mirmohseni A., Azizi M., Dorraji M.S.S. (2020). Cationic graphene oxide nanosheets intercalated with polyaniline nanofibers: A promising candidate for simultaneous anticorrosion, antistatic, and antibacterial applications. Prog. Org. Coat..

[63] De Almeida N.R., Han Y., Perez J., Kirkpatrick S., Wang Y., Sheridan M.C. (2019). Design, Synthesis, and Nanostructure-Dependent Antibacterial Activity of Cationic Peptide Amphiphiles. ACS Appl. Mater. Interfaces.

[64] Upadhyay R.K., Waghmare P.R. (2020). Eco-friendly preparation of superhydrophobic copper surfaces for oil/water separation. Environ. Chem. Lett..

[65] Maffei A., Michieli N., Brun P., Zamuner A., Zaggia A., Roso M., Kalinic B., Falzacappa E.V., Scopece P., Gross S. (2020). An atmospheric pressure plasma jet to tune the bioactive peptide coupling to polycaprolactone electrospun layers. Appl. Surf. Sci..

[66] Hesari S.M., Ghorbani F., Ghorbani F., Zamanian A., Khavandi A. (2021). Plasma surface modification technique–induced gelatin grafting on bio-originated polyurethane porous matrix: Physicochemical and in vitro study. Polym. Polym. Compos..

[67] Ghorbani F., Zamanian A., Aidun A. (2020). Conductive electrospun polyurethane-polyaniline scaffolds coated with poly(vinyl alcohol)-GPTMS under oxygen plasma surface modification. Mater. Today Commun..

[68] Hamdan N., Yamin A., Hamid S.A., Khodir W.K.W.A., Guarino V. (2021). Functionalized Antimicrobial Nanofibers: Design Criteria and Recent Advances. J. Funct. Biomater..

[69] Asadian M., Dhaenens M., Onyshchenko I., Waele S.D., Declercq H., Cools P., Devreese B., Deforce D., Morent R., Geyter N.D. (2018). Plasma functionalization of PCL nanofibers changes protein interactions with cells resulting in increased cell viability. ACS Appl. Mater. Interfaces.

[70] Kooshki H., Ghollasi M., Halabian R., Kazemi N.M. (2019). Osteogenic differentiation of preconditioned bone marrow mesenchymal stem cells with lipopolysaccharide on modified poly-l-lactic-acid nanofibers. J. Cell. Physiol..

[71] Habte A.T., Ayele D.W. (2019). Synthesis and Characterization of Reduced Graphene Oxide (rGO) Started from Graphene Oxide (GO) Using the Tour Method with Different Parameters. Adv. Mater. Sci. Eng..

[72] Ashraf M.A., Liu Z., Peng W.-X., Jermsittiparsert K., Hosseinzadeh G., Hosseinzadeh R. (2019). Combination of sonochemical and freeze-drying methods for synthesis of graphene/Ag-doped TiO2 nanocomposite: A strategy to boost the photocatalytic performance via well distribution of nanoparticles between graphene sheets. Ceram. Int..

[73] Doostmohammadi M., Forootanfar H., Shakibaie M., Torkzadeh-Mahani M., Rahimi H., Jafari E., Ameri A., Ameri A. (2021). Polycaprolactone/gelatin electrospun nanofibres containing biologically produced tellurium nanoparticles as a potential wound dressing scaffold: Physicochemical, mechanical, and biological characterisation. IET Nanobiotechnol..

[74] Dulnik J., Kołbuk D., Denis P., Sajkiewicz P. (2018). The effect of a solvent on cellular response to PCL/gelatin and PCL/collagen electrospun nanofibres. Eur. Polym. J..

[75] Borges-Vilches J., Unalan I., Fernandez K., Boccaccini A.R. (2022). Fabrication of biocompatible electrospun poly(ε-caprolactone)/gelatin nanofibers loaded with Pinus radiata bark extracts for wound healing applications. Polymers.

[76] Dufay M., Jimenez M., Degoutin S. (2020). Effect of Cold Plasma Treatment on Electrospun Nanofibers Properties: A Review. ACS Appl. Bio Mater..

[77] Heidari M., Bahrami S.H., Ranjbar-Mohammadi M., Milan P.B. (2019). Smart electrospun nanof bers containing PCL/gelatin/graphene oxide for application in nerve tissue engineering. Mater. Sci. Eng. C.

[78] Abrigo M., Kingshott P., McArthur S.L. (2015). Bacterial response to different surface chemistries fabricated by plasma polymerization on electrospun nanofibers. Biointerphases.

[79] Linklater D.P., De Volder M., Baulin V.A., Werner M., Jessl S., Golozar M., Maggini L., Rubanov S., Hanssen E., Juodkazis S. (2018). High Aspect Ratio Nanostructures Kill Bacteria via Storage and Release of Mechanical Energy. ACS Nano.

[80] Elbourne A., Coyle V.E., Truong V.K., Sabri Y.M., Kandjani A.E., Bhargava S.K., Ivanova E.P., Crawford R.J. (2019). Multi-directional electrodeposited gold nanospikes for antibacterial surface applications. Nanoscale Adv..

[81] Xie Y., He Y., Chen X., Bu D., He X., Zhi M., Wang M. (2021). Relationship between mechano-bactericidal activity and nanoblades density on chemically strengthened glass. Nanotechnol. Rev..

[82] Perreault F., de Faria A.F., Nejati S., Elimelech M. (2015). Antimicrobial Properties of Graphene Oxide Nanosheets: Why Size Matters. ACS Nano.

[83] Yoo M.J., Park H.B. (2019). Effect of hydrogen peroxide on properties of graphene oxide in Hummers method. Carbon.

[84] Nanda S., Gaur A., Duchaniya R.K. (2020). Synthesis, properties and applications of graphene oxide: An overview. World Sci. News.

[85] Sun L. (2019). Structure and synthesis of graphene oxide. Chin. J. Chem. Eng..

[86] Nishina Y., Eigler S. (2020). Chemical and electrochemical synthesis of graphene oxide—A generalized view. Nanoscale.

[87] Al-Gaashani R., Najjar A., Zakaria Y., Mansour S., Atieh M.A. (2019). XPS and structural studies of high quality graphene oxide and reduced graphene oxide prepared by different chemical oxidation methods. Ceram. Int..

[88] Kang L., Zhao L., Yao S., Duan C. (2019). A new architecture of super-hydrophilic β-SiAlON/graphene oxide ceramic membrane for enhanced anti-fouling and separation of water/oil emulsion. Ceram. Int..

[89] Junaidi N.F.D., Othman N.H., Shahruddin M.Z., Alias N.H., Lau W.J., Ismail A.F. (2019). Effect of graphene oxide (GO) and polyvinylpyrollidone (PVP) additives on the hydrophilicity of composite polyethersulfone (PES) membrane. Malays. J. Fundam. Appl. Sci..

[90] Lim J.Y., Mubarak N., Abdullah E., Nizamuddin S., Khalid M. (2018). Inamuddin Recent trends in the synthesis of graphene and graphene oxide based nanomaterials for removal of heavy metals—A review. J. Ind. Eng. Chem..

[91] Olorunkosebi A.A., Eleruja M.A., Adedeji A.V., Olofinjana B., Fasakin O., Omotoso E., Oyedotun K.O., Ajayi E.O.B., Manyala N. (2021). Optimization of graphene oxide through various Hummers’ methods and comparative reduction using green approach. Diam. Relat. Mater..

[92] Turkaslan E.B., Aydin F.M. (2020). Optimizing parameters of graphene derivatives synthesis by modified improved Hummers. Math. Methods Appl. Sci..

[93] Antonio R.D.S., Guerra A.C.S., Andrade M.B.D., Nishi L., Baptista A.T.A., Bergamasco R., Vieira A.M.S. (2020). Application of graphene nanosheet oxide for atrazinw adsorption in aqueous solution: Synthesis, material characterization, and comprehension of the adsorption mechanism. Environ. Sci. Pollut. Res..

[94] Aliyev E., Filiz V., Khan M.M., Lee Y.J., Abetz C., Abetz V. (2019). Structural Characterization of Graphene Oxide: Surface Functional Groups and Fractionated Oxidative Debris. Nanomaterials.

[95] Yu B., Wang K., Hu Y., Nan F., Pu J., Zhao H., Ju P. (2018). Tribological properties of synthethic base oil containing polyhedral oligomeric silsesquioxane grafted graphene oxide. RSC Adv..

[96] Sengupta I., Bhattacharya P., Talukdar M., Neogi S., Pal S.K., Chakraborty S. (2019). Bactericidal effect of graphene oxide and reduced graphene oxide: Influence of shape of bacteria. Colloid Interface Sci. Commun..

[97] Montes-Duarte G.G., Tostado-Blázquez G., Castro K.L.S., Araujo J.R., Achete C.A., Sánchez-Salas J.L., Campos-Delgado J. (2021). Key parameters to enhance the antibacterial effect of graphene oxide in solution. RSC Adv..

[98] Surekha G., Krishnaiah K.V., Ravi N., Suvarna R.P. (2020). FTIR, Raman and XRD analysis of graphene oxide films prepared by modified Hummers method. J. Phys. Conf. Ser..

[99] Muniyalakshmi M., Sethuraman K., Silambarasan D. (2020). Synthesis and characterization of graphene oxide nanosheets. Mater. Today: Proc..

[100] Alkhouzaam A., Qiblawey H., Khraisheh M., Atieh M., Al-Ghouti M. (2020). Synthesis of graphene oxides particle of high oxidation degree using a modified Hummers method. Ceram. Int..

[101] Muzyka R., Drewniak S., Pustelny T., Chrubasik M., Gryglewicz G. (2018). Characterization of Graphite Oxide and Reduced Graphene Oxide Obtained from Different Graphite Precursors and Oxidized by Different Methods Using Raman Spectroscopy. Materials.

[102] Lee A.Y., Yang K., Anh D.N., Park C., Lee M.S., Lee G.T., Jeong M.S. (2021). Raman study of D * band in graphene oxide and its correlation with reduction. Appl. Surf. Sci..

[103] Su W., Kumar N., Krayev A., Chaigneau M. (2018). In situ topographical chemical and electrical imaging of carboxyl graphene oxide at the nanoscale. Nat. Commun..

[104] Dasari B.L., Morshed M., Nouri J.M., Brabazon D., Naher S. (2018). Mechanical properties of graphene oxide reinforced aluminium matrix composites. Compos. Part B Eng..

[105] Menazea A., Ahmed M. (2020). Synthesis and antibacterial activity of graphene oxide decorated by silver and copper oxide nanoparticles. J. Mol. Struct..

[106] Lu B.-Y., Zhu G.-Y., Yu C.-H., Chen G.-Y., Zhang C.-L., Zeng X., Chen Q.-M., Peng Q. (2021). Functionalized graphene oxide nanosheets with unique three-in-one properties for efficient and tunable antibacterial applications. Nano Res..

[107] Gea S., Situmorang S.A., Pasaribu N., Piliang A.F., Attaurrazaq B., Sari R.M., Pasaribu K.M., Goutianos S. (2022). Facile synthesis of ZnO–Ag nanocomposite supported by graphene oxide with stabilised band-gap and wider visible-light region for photocatalyst application. J. Mater. Res. Technol..

[108] Ghasemi A., Imani R., Yousefzadeh M., Bonakdar S., Solouk A., Fakhrzadeh H. (2019). Studying the Potential Application of Electrospun Polyethylene Terephthalate/Graphene Oxide Nanofibers as Electroconductive Cardiac Patch. Macromol. Mater. Eng..

[109] Coelho C.S., Estevinho N.B., Rocha F. (2021). Encapsulation in food industry with emerging electrohydrodynamic techniques: Electrospinning and electrospraying—A review. Food Chem..

[110] Ghorani B., Tucker N. (2015). Fundamentals of electrospinning as a novel delivery vehicle for bioactive compounds in food nanotechnology. Food Hydrocoll..

[111] Niemczyk-Soczynska B., Gradys A., Sajkiewicz P. (2020). Hydrophilic Surface Functionalization of Electrospun Nanofibrous Scaffolds in Tissue Engineering. Polymers.

[112] Sun C., Zeng X., Zheng S., Wang Y., Li Z., Zhang H., Nie L., Zhang Y., Zhao Y., Yang X. (2021). Bio-adhesive catechol-modified chitosan wound healing hydrogel dressings through glow discharge plasma technique. Chem. Eng. J..

[113] Meghdadi M., Atyabi S.-M., Pezeshki-Modaress M., Irani S., Noormohammadi Z., Zandi M. (2019). Cold atmospheric plasma as a promising approach for gelatin immobilization on poly(ε-caprolactone) electrospun scaffolds. Prog. Biomater..

[114] Meghdadi M., Pezeshki-Modaress M., Irani S., Atyabi S.M., Zandi M. (2019). Chondroitin sulfate immobilized PCL nanofibers enhance chondrogenic differentiation of mesenchymal stem cells. Int. J. Biol. Macromol..

[115] Ren K., Wang Y., Sun T., Yue W., Zhang H. (2017). Electrospun PCL/gelatin composite nanofiber structures for effective guided bone regeneration membranes. Mater. Sci. Eng. C.

[116] Aidun A., Firoozabady S., Moharrami A., Ahmad A., Haghighpour N., Bonakdar S., Faghihi S. (2019). Graphene oxide incorporated polycaprolcatone/chitosan/gelatin electrospun scaffold: Enhdanced osteogenic properties for bone tissue engineering. Artif. Organs.

[117] Georgiev A., Stoilova A., Dimov D., Yordanov D., Zhivkov I., Weiter M. (2019). Synthesis and photochromic properties of some N-phthalimide azo-azomethine dyes. A DFT quantum mechanical calculations on imine-enamine tautomerism and trans-cis photoisomerization. Spectrochim. Acta Part A Mol. Biomol. Spectrosc..

[118] Jin M., Yin S.-F., Yang S.-D. (2020). Bismuth(III)-Catalyzed Sequential Enamine–Imine Tautomerism/2-Aza-Cope Rearrangement of Stable β-Enaminophosphonates: One-Pot Synthesis of β-Aminophosphonates. Org. Lett..

[119] Martínez R.F., Matamoros E., Cintas P., Palacios J.C. (2020). Imine or Enamine? Insights and Predictive Guidelines from the Electronic Effect of Substituents in H-Bonded Salicylimines. J. Org. Chem..

[120] Asadian M., Chan K.V., Egghe T., Onyshchenko Y., Grande S., Declercq H., Cools P., Morent R., De Geyter N. (2021). Acrylic acid plasma polymerization and post-plasma ethylene diamine grafting for enhanced bone marrow mesenchymal stem cell behaviour on polycaprolactone nanofibers. Appl. Surf. Sci..

[121] Wang Y., Guo Z., Qian Y., Zhang Z., Lyu L., Wang Y., Ye F. (2019). Study on the Electrospinning of Gelatin/Pullulan Composite Nanofibers. Polymers.

[122] Ghasemi-Mobarakeh L., Prabhakaran M.P., Morshed M., Nasr-Esfahani M.H., Ramakrishna S. (2008). Electropsun poly(ɛ-caprolactone)/gelatin nanofiberous scaffolds for nerve tissue engineering. Biomaterials.

[123] Khodir W.K.W.A., Hamid S.A., Yusof M.R., Cruz-Maya I., Guarino V. (2022). Electrospun Sulfonatocalix[4]arene Loaded Blended Nanofibers: Process Optimization and In Vitro Studies. Pharmaceutics.

[124] Demirci N., Demirel M., Dilsiz N. (2014). Surface Modification of PVC Film with Allylamine Plasma Polymers. Adv. Polym. Technol..

[125] Akbari S., Eslahi N., Kish M.H. (2017). Evaluation of hydrophilic properties of acrylonitrile/acrylic acid copolymer films dendrigrafted with citric acid. Polyolefins J..

[126] Kruger-Genge A., Braune S., Walter M., Krengel M., Kratz K., Kupper J.H. (2018). Influence of different surface treatments of poly(n -butyl acrylate) networks on fibroblasts adhesion, morphology and viability. Clin. Hemorheol. Microcirc..

[127] Park J.K., Pham-Nguyen O.-V., Yoo H.S. (2020). Coaxial Electrospun Nanofibers with Different Shell Contents to Control Cell Adhesion and Viability. ACS Omega.

[128] Lee S.J., Kim H.-J., Heo M., Lee H.-R., Choi E.-J., Kim H., Lee D., Reis R.L., Do S.H., Kwon I.K. (2019). In vitro and in vivo assessments of an optimal polyblend composition of polycaprolactone/gelatin nanofibrous scaffolds for Achilles tendon tissue engineering. J. Ind. Eng. Chem..

[129] Shi R., Geng H., Gong M., Ye J., Wu C., Hu X. (2018). Long-acting and broad-spectrum antimicrobial electrospun poly(ε-caprolactone)/gelatin micro/nanofibers for wound dressing. J. Colloid Interface Sci..

[130] Hamdan N., Darnis D.S., Khodir W.K.W.A. (2021). In Vitro Evaluation of Crosslinked Polyvinyl Alcohol/Chitosan—Gentamicin Sulfate Electrospun Nanofibers. Malays. J. Chem..

[131] Kharaghani D., Gitigard P., Ohtani H., Kim K.O., Ullah S. (2019). Design and characterization of dual drug delivery based on in-situ assembled PVA / PAN core-shell nanofibers for wound dressing application. Sci. Rep..

[132] Shanesazzadeh E., Kadivar M., Fathi M. (2018). Production and characterization of hydrophilic and hydrophobic sunflower protein isolate nanofibers by electrospinning method. Int. J. Biol. Macromol..

[133] Yang Z., Li X., Si J., Cui Z., Peng K. (2019). Morphological, mechanical and thermal properties of poly(lactic acid) (pla)/cellulose nanofibrils (CNF) composites nanofiber for tissue engineering. J. Wuhan Univ. Technol. Mater. Sci. Ed..

[134] Aynali F., Balci H., Doganci E., Bulus E. (2021). Production and characterization of non-leaching antimicrobial and hydrophilic polycaprolactone based nanofiber mats. Eur. Polym. J..

[135] Aboamera N.M., Mohamed A., Salama A., Osman T., Khattab A. (2017). Characterization and mechanical properties of electrospun cellulose acetate/graphene oxide composite nanofibers. Mech. Adv. Mater. Struct..

[136] Lin M., Liu Y., Gao J., Wang D., Xia D., Liang C., Li N., Xu R. (2022). Synergistic Effect of Co-Delivering Ciprofloxacin and Tetracycline Hydrochloride for Promoted Wound Healing by Utilizing Coaxial PCL/Gelatin Nanofiber Membrane. Int. J. Mol. Sci..

[137] Wang Z., Wang H., Xiong J., Li J., Miao X., Lan X., Liu X., Wang W., Cai N., Tang Y. (2021). Fabrication and in vitro evaluation of PCL/gelatin hierarchical scaffolds based on melt electrospinning writing and solution electrospinning for bone regeneration. Mater. Sci. Eng. C.

[138] Asadi H., Ghaee A., Nourmohammadi J., Mashak A. (2019). Electrospun zein/graphene oxide nanosheet composite nanofibers with controlled drug release as antibacterial wound dressing. Int. J. Polym. Mater. Polym. Biomater..

[139] Wang L., Chen Y., Lin L., Wang H., Huang X., Xue H., Gao J. (2019). Highly stretchable, anti-corrosive and wearable strain sensors based on the PDMS/CNTs decorated elastomer nanofiber composite. Chem. Eng. J..

[140] El-Aassar M., El-Kady M., Hassan H.S., Al-Deyab S.S. (2015). Synthesis and characterization of surface modified electrospun poly (acrylonitrile-co-styrene) nanofibers for dye decolorization. J. Taiwan Inst. Chem. Eng..

[141] Pourjavaher S., Almasi H., Meshkini S., Pirsa S., Parandi E. (2017). Development of a colorimetric pH indicator based on bacterial cellulose nanofibers and red cabbage (*Brassica oleraceae*) extract. Carbohydr. Polym..

[142] Ma G., Qi J., Cui Q., Bao X., Gao D., Xing C. (2020). Graphene Oxide Composite for Selective Recognition, Capturing, Photothermal Killing of Bacteria over Mammalian Cells. Polymers.

[143] Yang F., Feng Y., Fan X., Zhang M., Wang C., Zhao W., Zhao C. (2019). Biocompatible graphene-based nanoagent with NIR and magnetism dual-responses for effective bacterial killing and removal. Colloids Surf. B: Biointerfaces.

[144] Begum S., Pramanik A., Davis D., Patibandla S., Gates K., Gao Y., Ray P.C. (2020). 2D and Heterostructure Nanomaterial Based Strategies for Combating Drug-Resistant Bacteria. ACS Omega.

[145] Carniello V. (2018). Response of Staphylococcus aureus to mechanical and chemical stresses. Rijksuniversitieit Gronigen.

[146] Carniello V., Peterson B.W., Sjollema J., Busscher H.J., Van Der Mei H.C. (2018). Surface enhanced fluorescence and nanoscopic cell wall deformation in adhering Staphylococcus aureus upon exposure to cell wall active and non-active antibiotics. Nanoscale.

[147] Homaeigohar S., Boccaccini A.R. (2020). Antibacterial biohybrid nanofibers for wound dressings. Acta Biomater..

[148] Gratzl G., Paulik C., Hild S., Guggenbichler J.P., Lackner M. (2014). Antimicrobial activity of poly(acrylic acid) block copolymers. Mater. Sci. Eng. C.

[149] Gratzl G., Walkner S., Hild S., Hassel A.W., Weber H.K., Paulik C. (2015). Mechanistic approaches on the antibacterial activity of poly(acrylic acid) copolymers. Colloids Surf. B Biointerfaces.

[150] Sanni O., Chang C., Anderson D.G., Langer R., Davies M.C., Williams P.M., Williams P., Alexander M.R., Hook A.L. (2015). Bacterial Attachment to Polymeric Materials Correlates with Molecular Flexibility and Hydrophilicity. Adv. Healthc. Mater..

[151] Jiang Y., Ma D., Ji T., Sameen D.E., Ahmed S., Li S., Liu Y. (2020). Long-Term Antibacterial Effect of Electrospun Polyvinyl Alcohol/Polyacrylate Sodium Nanofiber Containing Nisin-Loaded Nanoparticles. Nanomaterials.

[152] Epa V.C., Hook A., Chang C.-Y., Yang J., Langer R., Anderson D.G., Williams P., Davies M., Alexander M., Winkler D.A. (2014). Modelling and Prediction of Bacterial Attachment to Polymers. Adv. Funct. Mater..

[153] Hook A., Chang C.-Y., Yang J., Atkinson S., Langer R., Anderson D.G., Davies M., Williams P., Alexander M.R. (2013). Discovery of Novel Materials with Broad Resistance to Bacterial Attachment Using Combinatorial Polymer Microarrays. Adv. Mater..

[154] Abrigo M., Kingshott P., McArthur S.L. (2015). Electrospun fiber diameter influencing bacterial attachment, proliferation, and growth. ACS Appl. Mater. Interfaces.

[155] Mukherjee I., Ghosh A., Bhadury P., De P. (2017). Side-Chain Amino Acid-Based Cationic Antibacterial Polymers: Investigating the Morphological Switching of a Polymer-Treated Bacterial Cell. ACS Omega.

[156] Hasan N., Cao J., Lee J., Hlaing S.P., Oshi M.A., Naeem M. (2019). Bacteria-targeted clindamycin loaded polymeris nanoparticles: Effect of surface charge on nanoparticle adhesion to MRSA, antibacterial activity, and wound healing. Pharmaceutics.

[157] Wu J., Zhao S., Xu S., Pang X., Cai G., Wang J. (2018). Acidity-triggered charge-reversible multilayers for construction of adaptive surfaces with switchable bactericidal and bacteria-repelling functions. J. Mater. Chem. B.

[158] Peer P., Janalikova M., Sedlarikova J., Pleva P., Filip P., Zelenkova J., Siskova A.O. (2021). Antibacterial Filtration Membranes Based on PVDF-*co*-HFP Nanofibers with the Addition of Medium-Chain 1-Monoacylglycerols. ACS Appl. Mater. Interfaces.

[159] Siddiqi K.S., Husen A., Rao R.A.K. (2018). A review on biosynthesis of silver nanoparticles and their biocidal properties. J. Nanobiotechnol..

[160] Černochová P., Blahová L., Medalová J., Nečas D., Michlíček M., Kaushik P., Přibyl J., Bartošíková J., Manakhov A., Bačáková L. (2020). Cell type specific adhesion to surfaces functionalised by amine plasma polymers. Sci. Rep..

[161] Huzum B., Puha B., Necoara R.M., Gheorghevici S., Puha G., Filip A., Sirbu P.D., Alexa O. (2021). Biocompatibility assessment of biomaterials used in orthopedic devices: An overview (Review). Exp. Ther. Med..

[162] Saracino E., Cirillo V., Marrese M., Guarino V., Benfenati V., Zamboni R., Ambrosio L. (2021). Structural and functional properties of astrocytes on PCL based electrospun fibres. Mater. Sci. Eng. C.

[163] Cruz-Maya I., Varesano A., Vineis C., Guarino V. (2020). Comparative Study on Protein-Rich Electrospun Fibers for in Vitro Applications. Polymers.

[164] Cruz-Maya I., Guarino V., Almaguer-Flores A., Alvarez-Perez M.A., Varesano A., Vineis C. (2019). Highly polydisperse keratin rich nanofibers: Scaffold design and in vitro characterization. J. Biomed. Mater. Res. Part A.

[165] Cirillo V., Guarino V., Alvarez-Perez M.A., Marrese M., Ambrosio L. (2014). Optimization of fully aligned bioactive electrospun fibers for “in vitro” nerve guidance. J. Mater. Sci. Mater. Med..

[166] Zavan B., Gardin C., Guarino V., Rocca T., Maya I.C., Zanotti F., Ferroni L., Brunello G., Chachques J.-C., Ambrosio L. (2021). Electrospun PCL-Based Vascular Grafts: In Vitro Tests. Nanomaterials.

[167] Zhang L., Xu B., Wang X. (2016). Cholesterol Extraction from Cell Membrane by Graphene Nanosheets: A Computational Study. J. Phys. Chem. B.

